# Inhibition of DDR1 potentiates carbon ion radiotherapy by promoting ferroptosis and immunogenic death in head and neck squamous cell carcinoma

**DOI:** 10.1186/s12967-025-07062-5

**Published:** 2025-09-24

**Authors:** Wei Hu, Qingting Huang, Li Chen, Shikai Geng, Haojiong Zhang, Huaiyuan Chen, Xingyu Liu, Jingqin Zhong, Fengtao Su, Chunlin Shao, Lin Kong

**Affiliations:** 1 Department of Radiation Oncology, Shanghai Proton and Heavy Ion Center, Fudan University Cancer Hospital, Shanghai, 201315 China; 2 Shanghai Key Laboratory of Radiation Oncology, Shanghai, 201315 China; 3 Shanghai Engineering Research Center of Proton and Heavy Ion Radiation Therapy, Shanghai, 201315 China; 4 Department of Research and Development, Shanghai Proton and Heavy Ion Center, Fudan University Cancer Hospital, Shanghai, 201315 China; 5 Cancer Institute, Fudan University Shanghai Cancer Center, Shanghai Medical College, Fudan University, Shanghai, China; 6 Institute of Radiation Medicine, Shanghai Medical College, Fudan University, Shanghai, China

**Keywords:** Carbon ion radiotherapy, Discoidin domain receptor 1, Ferroptosis, Immunogenic cell death, PI3K/Akt/mTOR signaling, Head and neck squamous cell carcinoma

## Abstract

**Background:**

Carbon ion radiotherapy (CIR) has emerged as a promising therapeutic modality for photon-resistant malignancies due to its unique physical depth-dose distribution and enhanced radiobiological effectiveness. Nevertheless, treatment resistance persists in certain recurrent or refractory head and neck squamous cell carcinoma (HNSCC) cases, underscoring the need for novel combinatorial strategies. Here, we demonstrated the sensitizing effect of targeting discoidin domain receptor 1 (DDR1) in HNSCC for CIR.

**Methods:**

MOC1 and and Cal27 cell lines along with tumor-bearing C57BL/6 mice were used for in vitro and in vivo studies. DDR1 was knocked down via lentivirus. Cell viability and proliferation were assessed by CCK-8 and colony formation assays. Immunogenicity and tumor-infiltrating lymphocytes were measured via flow cytometry and immunofluorescence. Tumor suppression mechanisms were investigated using RNA sequencing and bioinformatics. Ferroptosis markers (lipid peroxidation, iron, ROS) were detected using MDA, BODIPY 581/591 C11, FerroOrange, and DCFH-DA probes. Upstream ferroptosis mechanisms were analyzed by Western blot, co-immunoprecipitation, key molecule modulator administration, and SCD1 overexpression.

**Results:**

We demonstrated that targeting DDR1 potentiated CIR by triggering ferroptosis-mediated immunogenic cell death, which in turn enhanced antitumor immunity. Mechanistically, DDR1 sustained tumor cell survival by forming 14–3-3-mediated assembly of a DDR1/14–3-3/Akt ternary complex, thereby activating the Akt/mTORC1/SREBP1/SCD1 axis to promote monounsaturated fatty acid (MUFA) biosynthesis and suppress ferroptosis. Silencing DDR1 disrupted this complex, alleviating MUFA-mediated ferroptosis inhibition and subsequently increasing tumor immunogenicity. This immunogenic shift facilitated CD8 + T cell infiltration and cytotoxicity, amplifying CIR-induced tumor suppression. Furthermore, pharmacological inhibition of DDR1 using the small-molecule inhibitor 7rh recapitulated these effects, demonstrating potent anti-proliferative and ferroptosis-inducing capabilities, enhancing CIR sensitivity to better control tumor progression.

**Conclusions:**

Our findings positioned DDR1 targeting as a therapeutic strategy to potentiate CIR through immunogenic ferroptosis induction in HNSCC.

**Supplementary Information:**

The online version contains supplementary material available at 10.1186/s12967-025-07062-5.

## Introduction

Head and neck squamous cell carcinoma (HNSCC), a malignancy originating from the mucosal epithelium of the oral cavity, oropharynx, hypopharynx, or larynx, ranks as the sixth most common cancer globally and exhibits pronounced tumor heterogeneity [1–3]. Radiotherapy (RT) remains a cornerstone in the management of HNSCC, offering organ preservation advantages for both curative and adjuvant purposes. Early-stage HNSCC patients treated with definitive RT can achieve outcomes comparable to radical surgery while preserving anatomical integrity [4]. However, approximately 70% of HNSCC patients present with locally advanced disease (stage III-IV) at initial diagnosis. Despite aggressive chemoradiotherapy, 15–50% of these patients experience locoregional recurrence. For recurrent cases who have undergone previous radiotherapy, re-irradiation efficacy remains suboptimal, with 5-year survival rates stagnating at 10–30% [5, 6]. These limitations underscore the urgent need for innovative therapeutic strategies to overcome radioresistance and improve survival outcomes in HNSCC.

Carbon ion radiotherapy (CIR), an advanced form of particle therapy, has gained prominence as a promising treatment modality owing to its distinct physical and biological advantages. The characteristic "Bragg peak" energy deposition profile of carbon ions allows for precise tumor targeting while sparing surrounding normal tissues from excessive radiation exposure. As a high linear energy transfer (LET) radiation type, carbon ions generate complex DNA double-strand breaks (DSBs) with diminished reliance on oxygen availability and cell cycle phase, which effectively counteracts intrinsic radioresistance in hypoxic or quiescent tumor populations [7]. Supporting its clinical potential, our preliminary data in HNSCC demonstrate favorable outcomes—treatment-naïve patients receiving combined CIR achieved a 3-year local control rate of 83.5%, while re-irradiation with carbon ions in recurrent cases yielded 1-year survival and local control rates of 95.9% and 84.9%, respectively [8, 9]. Despite these advances, the 2-year local control rates for carbon ion re-irradiation alone remain suboptimal (40–60%) [9, 10], underscoring the critical need for combined therapeutic strategies to enhance treatment efficacy.

The advent of immune checkpoint inhibitors (ICIs), particularly PD-1/PD-L1 inhibitors, has revolutionized oncology. Preclinical and early clinical studies demonstrated a synergistic effect between photon radiotherapy and ICIs through enhanced tumor antigen release and immune activation [11–14]. However, results from a multicenter randomized prospective clinical study showed that the combination of photon radiotherapy and the ICI pembrolizumab did not significantly improve the tumor control and survival of locally advanced HNSCC [15], underscoring the need for optimized radio-immunotherapy strategies. Intriguingly, emerging evidence indicated that high-LET carbon ions might surpass photons in immunomodulatory capacity. By inducing immunogenic cell death (ICD), promoting tumor-associated antigen release, diversifying T-cell receptor repertoires, and reprogramming the immunosuppressive tumor microenvironment (TME), CIR could potentiate systemic antitumor immunity and amplify immunotherapy efficacy [16–19]. This paradigm positions CIR and immunotherapy combination as a compelling therapeutic avenue for refractory or recurrent HNSCC.

Concurrently, the identification of novel molecular targets to optimize treatment responses remains critical. Discoidin Domain Receptor 1 (DDR1), a collagen receptor with tyrosine kinase activity, has garnered attention as a regulator of tumor-stroma interactions and immune evasion [20, 21]. DDR1 activation via cleaved collagen I binding triggered NF-κB-NRF2 signaling to enhance macropinocytosis and mitochondrial biogenesis, fostering tumor progression in pancreatic cancer models [22]. Notably, the DDR1 extracellular domain mediated collagen fiber remodeling to restrict immune infiltration in breast cancer, while DDR1 inhibition promoted cytotoxic lymphocyte recruitment and tumor suppression [23]. Besides, the abnormal increase of DDR1 level correlated negatively with pro-inflammatory factors across multiple malignancies [24, 25], positioning DDR1 as a potential immunotherapeutic target. However, the tumor-suppressive effect and immune response resulting from the combined treatment of targeting DDR1 and CIR have not been elucidated, particularly in HNSCC.

Given these knowledge gaps, this study aims to investigate the combinatorial effects of DDR1 inhibition and CIR on tumor cell eradication and immune regulation in HNSCC, and explored the mechanisms underlying their antitumor efficacy, involved in AKT/mTOR signaling and immunogenic ferroptosis pathway. This work will establish preclinical evidence for a novel therapeutic paradigm with transformative potential in HNSCC management.

## Materials and methods

### Cell culture

The murine oral squamous cell carcinoma cell line MOC1 (catalog #CTCC-001–0916, Zhejiang Meisen Cell Technology Co., Ltd, China) was cultured in DMEM/F-12 medium (Gibco) supplemented with 10% fetal bovine serum (FBS; Gibco), 10 μg/mL insulin (Sigma-Aldrich), 10 ng/mL epidermal growth factor (EGF; PeproTech), 80 ng/mL hydrocortisone (Sigma-Aldrich), and 1% penicillin–streptomycin (Gibco). The human tongue squamous cell carcinoma cell line Cal27 (catalog #CTCC-003–0022, Zhejiang Meisen Cell Technology Co., Ltd, China) was maintained in DMEM (Gibco) supplemented with 10% FBS (Gibco) and 1% penicillin–streptomycin (Gibco). Both cell lines were maintained at 37 °C in a humidified 5% CO₂ incubator. The medium was refreshed every 2 days, and subculturing was performed at 90% confluency using 0.25% trypsin–EDTA (Gibco).

### Chemicals

Liproxstatin-1 (Lipro-1) was sourced from Selleck Chemicals (USA). Ferrostatin-1 (Fer-1), MK-2206, SC79, MHY1485, stearic acid (SA), and 7rh were procured from MedChemExpress (MCE, USA). Oleic acid (OA) was obtained from Sigma-Aldrich (USA). In vivo dosing regimens for Lipro-1 and 7rh are detailed in the "Mouse models and treatments" section. For in vitro studies, pharmacological agents were introduced 2–3 h pre-irradiation with distinct exposure durations: Fer-1 (5 μM) and MK-2206 (10 μM) remained in culture for 24 h, whereas 7rh (2 μM) was maintained for 12 h before withdrawal. SC79 (15 μM) and MHY1485 (10 μM) were removed 2 h post-irradiation. SA and OA (100 μM each) persisted until final analysis. Unless otherwise stated, all cellular endpoints were assessed at 48 h post-irradiation.

### Carbon ion radiotherapy

Carbon ion radiotherapy was administered through a heavy ion synchrotron accelerator (Siemens AG) at Shanghai Proton and Heavy Ion Center (SPHIC). In short, carbon ion beams were precisely delivered to the target with a homogeneous spread-out Bragg peak with the energy of 156.3 MeV/u (dose averaged LET 50 keV/μm). For cellular studies, culture vessels were mounted on a custom vertical stand orthogonally aligned to the beam axis. In animal experiments, tumor-bearing mice underwent anesthesia via intraperitoneal pentobarbital sodium (50 mg/kg) and were immobilized on a beam-perpendicular platform. A tailored protection device ensured localized irradiation to the target region while shielding surrounding tissues. It is worth noting that, except for the colony formation assay, we used 5 Gy as the irradiation dose for cells and animals. Pre-experiment of the colony formation assay revealed that 5 Gy irradiation resulted in negligible cell survival after 10 days. Consequently, the radiation dose was adjusted downward to 2.5 Gy specifically for colony formation assay. Furthermore, we also utilized a fractionated irradiation model in both parental and DDR1-knockdown cell lines for colony formation assay to investigate the radiosensitizing effects of DDR1 inhibition on carbon ion radiotherapy. This model employed fractionation schemes of 2.5 Gy × 2 fractions and 1 Gy × 5 fractions.

### Mouse models and treatments

Syngeneic tumor models were established by subcutaneous implantation of DDR1-knockdown or control MOC1 cells in the right hind flank of C57BL/6 mice (7–8 weeks old). Carbon ion irradiation was initiated when mean tumor volume reached 50–100 mm^3^. To evaluate ferroptosis-mediated tumor suppression, Lipro-1 (10 mg/kg; Selleck) was administered intraperitoneally daily from the date of radiotherapy until the end of the study. For 7rh intervention, mice received 7rh orally (25 mg/kg; MCE) every 48 h starting 7 days pre-irradiation until study termination. Tumor progression was monitored via serial caliper measurements, with volumes calculated as (length × width^2^)/2. Animals were humanely euthanized upon reaching the ethical endpoint or at predetermined timepoints for tissue analysis. All procedures complied with the guidelines of SPHIC Institutional Animal Care and Use Committee. The ethics committee allowed a maximum tumor size of 2000 mm^3^.

### Statistical analysis

Statistical analyses were conducted using GraphPad Prism v9.5.1 and SPSS v20.0. Group comparisons employed two-tailed unpaired t-tests (two groups) or one-way ANOVA (multiple groups), while longitudinal data (cell growth curves, tumor volumes) were assessed via two-way ANOVA. A significance threshold of *p* < 0.05 was applied, denoted by asterisks: *p* < 0.05 (*), < 0.01 (**), < 0.001 (***), and < 0.0001 (****).

The remaining materials and methods can be found in the Supplementary File.

## Results

### DDR1 overexpression correlates with immune evasion and poor prognosis in HNSCC

Analysis of data from The Cancer Genome Atlas-head and neck squamous cell carcinoma (TCGA-HNSC) dataset revealed a striking upregulation of DDR1 in tumor tissues compared to normal tissues (Supplementary Fig. 1A, B). ROC curve analysis further demonstrated the diagnostic utility of DDR1 in HNSCC, with an area under the curve (AUC) of 0.757 (Supplementary Fig. 1C). Critically, Kaplan–Meier survival analysis showed that patients with high DDR1 expression exhibited significantly shorter overall survival (Supplementary Fig. 1D), demonstrating the prognostic significance of DDR1.

Recent studies have emphasized the crucial role of DDR1 in driving immune evasion [20, 21, 23]. To dissect DDR1’s immunosuppressive function, we evaluated its correlation with immune-related markers. In the TCGA-HNSC dataset, DDR1 expression was found to be inversely correlated with the expression of CD8B, GZMB, IFNG, and IL12B, as well as scores for T cells, CD8 T cells, and cytotoxic cells (Supplementary Fig. 1E-L). Collectively, these findings could position DDR1 as a driver of tumor progression and therapy resistance by mediating immune evasion in HNSCC, thereby providing a rationale for targeting DDR1 to restore antitumor immunity and enhance therapeutic outcomes.

### DDR1 inhibition sensitizes HNSCC to CIR to suppress tumor malignancy

**Fig. 1:**
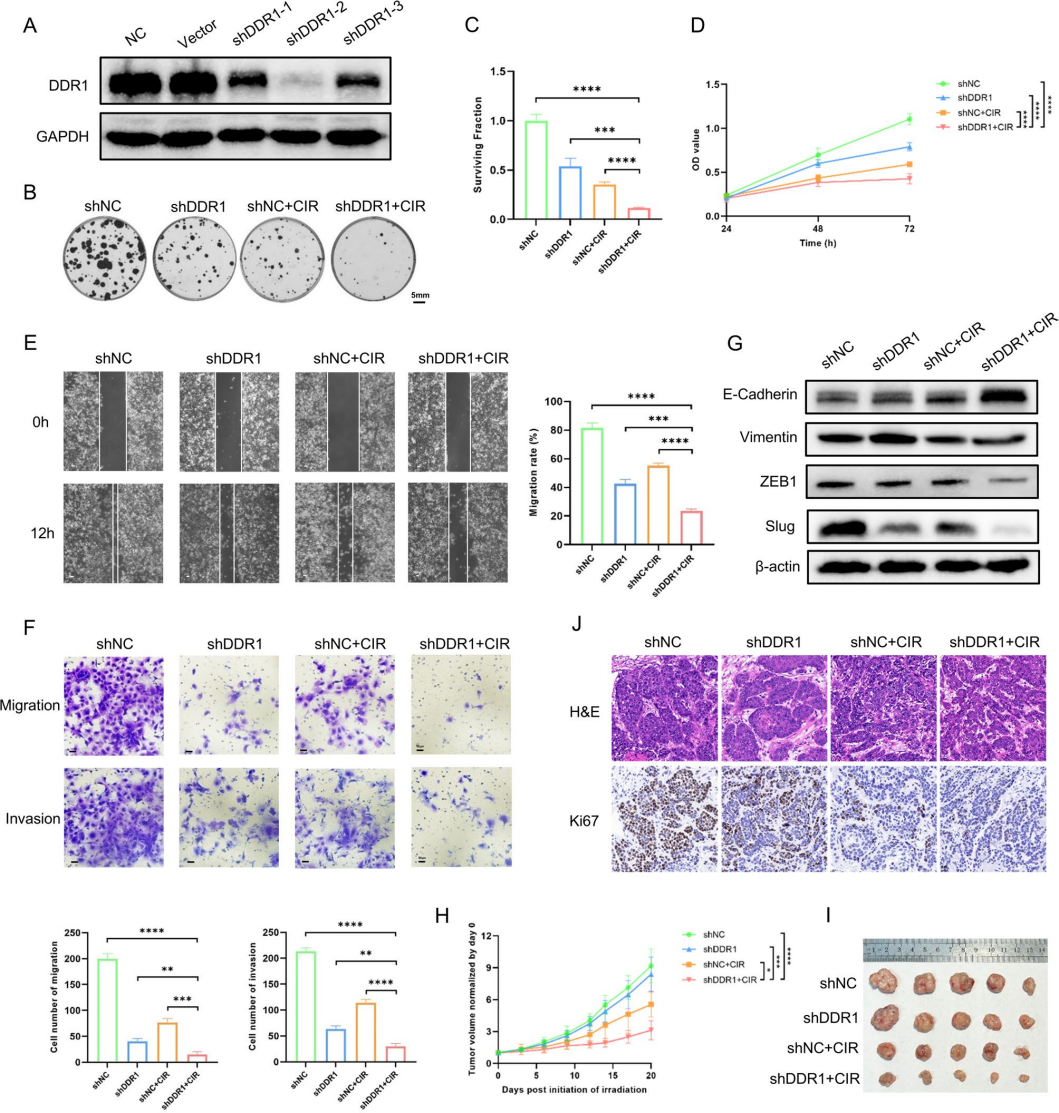
Antitumor effect of DDR1 inhibition combined with carbon ion radiotherapy in head and neck squamous cell carcinoma. **A** MOC1 cells were infected with three independent lentiviral particles for DDR1 knockdown. Western blot analysis of DDR1 protein levels in DDR1-knockdown and control cells. **B**, **C** Representative images or quantitative results of colony formation assay in MOC1 cells after DDR1 knockdown and carbon ion radiotherapy. Scale bar, 5 mm. **D** CCK-8 cell proliferation assay in MOC1 cells after DDR1 knockdown and carbon ion radiotherapy. **E** Representative images or quantitative results of wound healing in MOC1 cells after DDR1 knockdown and carbon ion radiotherapy. Scale bar, 50 μm. **F** Representative images or quantitative results of transwell migration and invasion in MOC1 cells after DDR1 knockdown and carbon ion radiotherapy. Scale bar, 50 μm. **G** Western blot analysis of EMT markers (E-cadherin, Vimentin, ZEB1 and Slug) in MOC1 cells after DDR1 knockdown and carbon ion radiotherapy. **H**, **I** Tumor growth and images of syngeneic MOC1 subcutaneous tumor models after DDR1 knockdown and carbon ion radiotherapy. N = 5 mice/group. **J** Representative images of H&E and immunohistochemistry staining of Ki67. Scale bar, 20 μm. * *p* < 0.05, *** *p* < 0.001, **** *p* < 0.0001

While CIR demonstrates promising efficacy in treating HNSCC, its limited effectiveness in refractory or recurrent cases underscores the need for combination strategies. To investigate whether DDR1 inhibition enhanced CIR sensitivity, we generated stable DDR1-knockdown MOC1 (shDDR1-2, hereinafter referred to as shDDR1) and Cal27 cells using lentiviral infection (Fig. 1A; Supplementary Fig. 3A). Consistent with the hypothesis of this study, colony formation and CCK-8 assays revealed that shDDR1 combined with CIR (shDDR1 + CIR) significantly reduced cell survival compared to the other three groups (Fig. 1B-D; Supplementary Fig. 2A-C, 3B-D). Furthermore, we found that the combination of DDR1 knockdown and CIR profoundly inhibited the migration, invasion, and epithelial-mesenchymal transition (EMT) of MOC1 cells (Fig. 1E-G).

Notably, the strong antitumor effect of inhibiting DDR1 combined with CIR was validated in vivo. In the syngeneic MOC1 subcutaneous tumor models, shDDR1 + CIR markedly suppressed tumor growth compared to monotherapy or control groups (Fig. 1H). Immunohistochemistry (IHC) further showed reduced Ki67 expression in shDDR1 + CIR tumors, indicating diminished proliferative activity (Fig. 1J). Collectively, these data indicated that targeting DDR1 was a promising therapeutic strategy to amplify CIR efficacy in HNSCC.

### Combined DDR1 inhibition and CIR promote immunogenic cell death

**Fig. 2:**
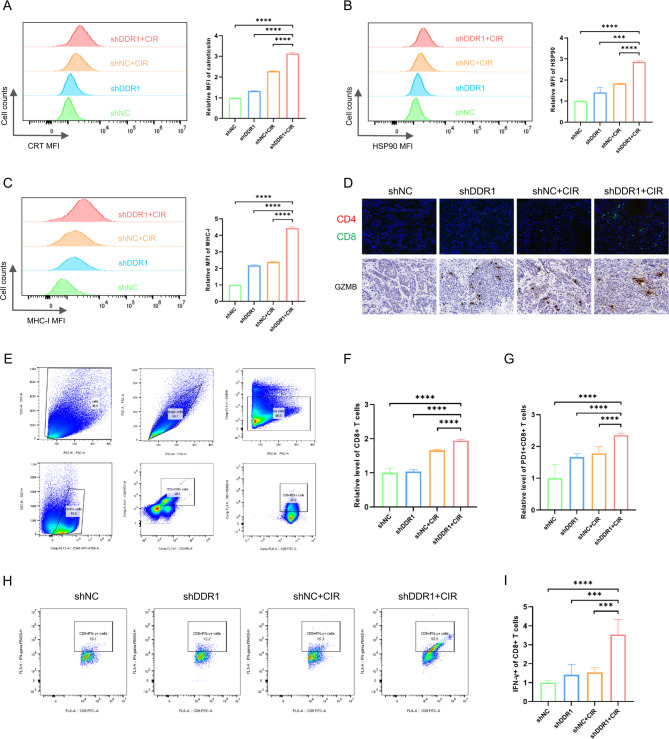
Inhibition of DDR1 combined with carbon ion radiotherapy induces immunogenic cell death. **A**–**C** Flow cytometry analysis of CRT, HSP90, and MHC-I expression levels on MOC1 cells after DDR1 knockdown and carbon ion radiotherapy. **D** Immunofluorescence assay of tumor-infiltrating CD4 + T cells (red) and CD8 + T cells (green), and immunohistochemistry staining of Granzyme B. Scale bar, 20 μm. **E** Gating strategy for flow cytometry analysis of T cell infiltration in tumor tissues. **F**, **G** Quantitative results of flow cytometry analysis of tumor-infiltrating CD8 + T cells and PD1 + CD8 + T cells. N = 6 mice/group. **H**, **I** Quantitative results of flow cytometry analysis of tumor‐infiltrating CD8 + IFN‐γ + cells. N = 6 mice/group. *** *p* < 0.001, **** *p* < 0.0001

Given the immunosuppressive role of DDR1 and its negative correlation with CD8 + T cell infiltration (Supplementary Fig. 1), we hypothesized that inhibition of DDR1 could further amplify CIR-induced antitumor immunity. On this basis, we first conducted in vitro cell experiments to investigate whether tumor cells underwent immunogenic cell death. Our findings revealed that the shDDR1 + CIR combination treatment significantly increased the exposure of damage-associated molecular patterns (DAMPs), such as calreticulin (CRT) and heat shock protein 90 (HSP90), on the surface of tumor cells (Fig. 2A, B; Supplementary Fig. 4A). Meanwhile, MHC-I on MOC1 cells and HLA-A, B, C on Cal27 cells exhibited a similar trend (Fig. 2C; Supplementary Fig. 4B, C), indicating that the combination of DDR1 inhibition and CIR significantly enhanced the immunogenicity of HNSCC cells.

Subsequently, we expanded our investigation into the combinatorial effects of DDR1 suppression and CIR on TME remodeling through in vivo experiment. Immunofluorescence analysis was conducted to analyze immune cell infiltration in murine tumor specimens and the results revealed pronounced enhancement of CD8 + T lymphocyte infiltration in the shDDR1 + CIR cohort (Fig. 2D). Flow cytometric quantification corroborated the finding, showing a substantial elevation in CD8 + T cell infiltration rates in the combination treatment group (Fig. 2E, F). Also, immunohistochemical and flow cytometric analyses demonstrated significantly elevated Granzyme B expression in tumor tissues, coupled with enhanced IFN-γ production capacity in CD8 + T cells (Fig. 2D, H), indicative of augmented cytotoxic potential in tumor-infiltrating immune cells. Notably, our investigation uncovered that concurrent DDR1 inhibition and CIR administration induced PD-1 molecule upregulation on CD8 + T cell surfaces (Fig. 2E, G), indicating a potential for achieving a better antitumor effect when combined with anti-PD-1 immune checkpoint inhibitors.

### Ferroptosis execution underlies the antitumor effects of DDR1 inhibition combined with CIR

**Fig. 3:**
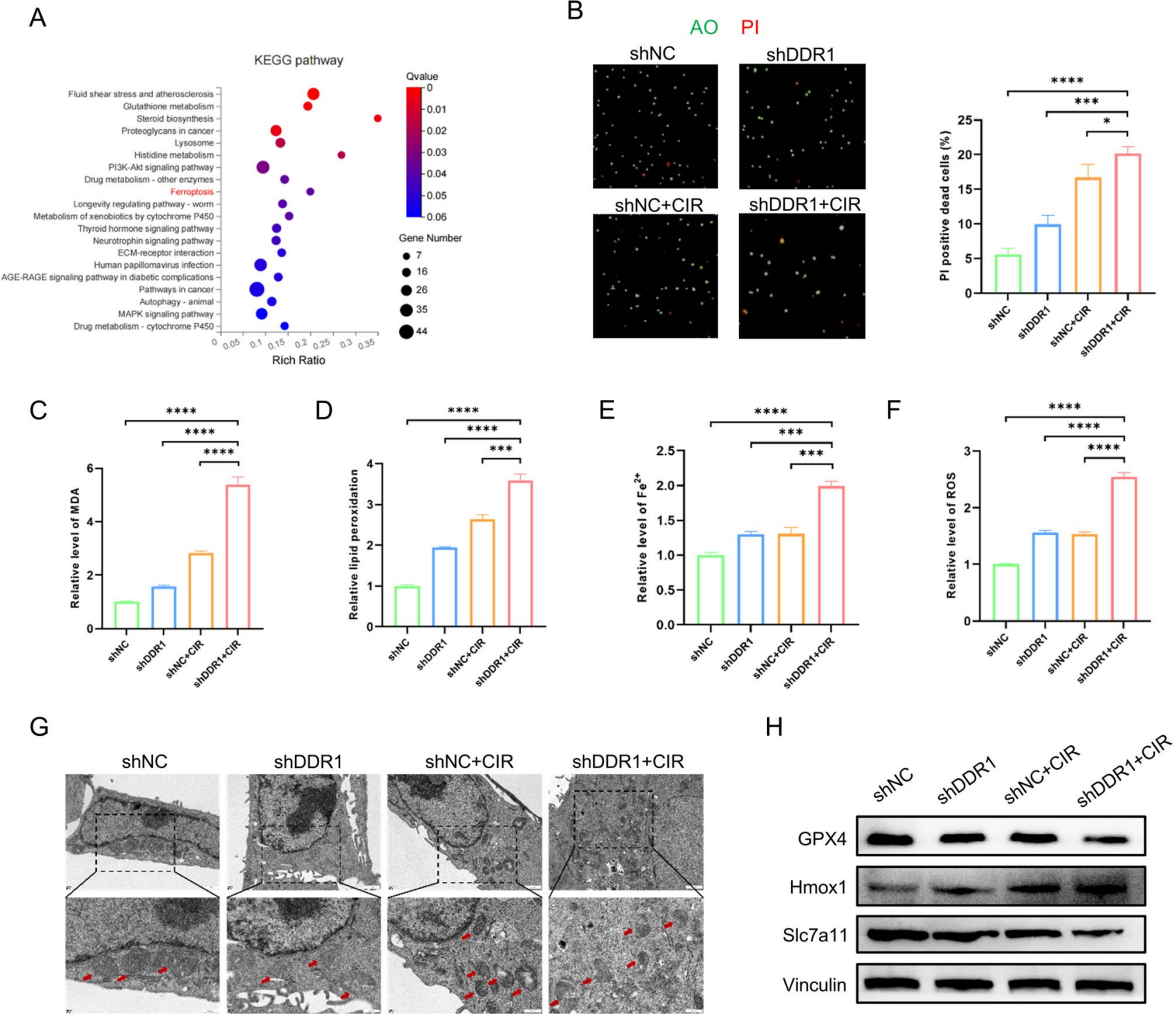
Inhibiting DDR1 sensitizes carbon ion radiotherapy by promoting ferroptosis. **A** KEGG pathway enrichment analysis of differentially expressed genes between the combined DDR1 knockdown and carbon ion radiotherapy group and other groups in MOC1 cells. **B** AO/PI staining to detect cell death in MOC1 cells after DDR1 knockdown and carbon ion radiotherapy. **C** MDA assay to measure lipid peroxidation levels in MOC1 cells after treatment. **D** BODIPY™ 581/591 C11 fluorescent probe to detect lipid peroxidation levels in MOC1 cells after treatment. **E** FerroOrange fluorescent probe to detect Fe^2+^ levels in MOC1 cells after treatment. **F** DCFH-DA fluorescent probe to detect ROS levels in MOC1 cells after treatment. **G** Transmission electron microscopy observation of mitochondrial changes (red arrows) in MOC1 cells after treatment. Scale bar, upper 1 μm, lower 500 nm. **H** Western blot analysis of GPX4, SLC7A11, and Hmox1 expression in MOC1 cells after treatment. * *p* < 0.05, *** *p* < 0.001, **** *p* < 0.0001

To elucidate the molecular mechanism underlying the therapeutic efficacy of shDDR1 + CIR combination therapy, we conducted RNA sequencing analysis. Comparative transcriptomic profiling between the shDDR1 + CIR group and control cohorts (shNC, shDDR1, shNC + CIR) identified 906 differentially expressed genes potentially mediating the antitumor advantage of the combinatorial treatment (Supplementary Fig. 6A). KEGG pathway enrichment analysis revealed involvement of the ferroptosis pathway among these candidate genes (Fig. 3A).

Ferroptosis, an iron-dependent programmed cell death modality driven by lipid peroxide accumulation, was subsequently investigated as a potential mechanism. Fluorescence co-staining with acridine orange/propidium iodide (AO/PI) demonstrated enhanced cellular mortality in the combination treatment group (Fig. 3B). Next, we found that the shDDR1 + CIR treatment induced maximal elevation of malondialdehyde (MDA) and lipid peroxidation compared to the other three groups (Fig. 3C, D; Supplementary Fig. 5A, B). Untargeted lipidomic profiling further revealed substantial enrichment of polyunsaturated fatty acid-containing phospholipids (PUFA-PLs) in the experimental cohort, particularly ferroptosis-associated phosphatidylethanolamines (PEs) bearing arachidonyl (20:4) or adrenyl (22:4) acyl chains (Supplementary Fig. 6B). Then, intracellular Fe^2+^ and reactive oxygen species (ROS) levels were detected and the results demonstrated the most pronounced accumulation of Fe^2^⁺ and ROS in shDDR1 + CIR-treated cells (Fig. 3E, F; Supplementary Fig. 5C, D). We also performed TEM analysis of cells, and found that the morphological changes of mitochondria in the shDDR1 + CIR group were the most obvious, including mitochondrial atrophy, increased membrane density, and reduced or even absent cristae (Fig. 3G, indicated by red arrows). Moreover, western blot analysis of several ferroptosis-related landmark molecules showed significant downregulation of GPX4 and SLC7A11 alongside upregulation of Hmox1 in the combination group (Fig. 3H; Supplementary Fig. 5E). Collectively, these findings suggested that combined DDR1 inhibition and CIR induced ferroptosis-driven tumor cell death in HNSCC.

**Fig. 4:**
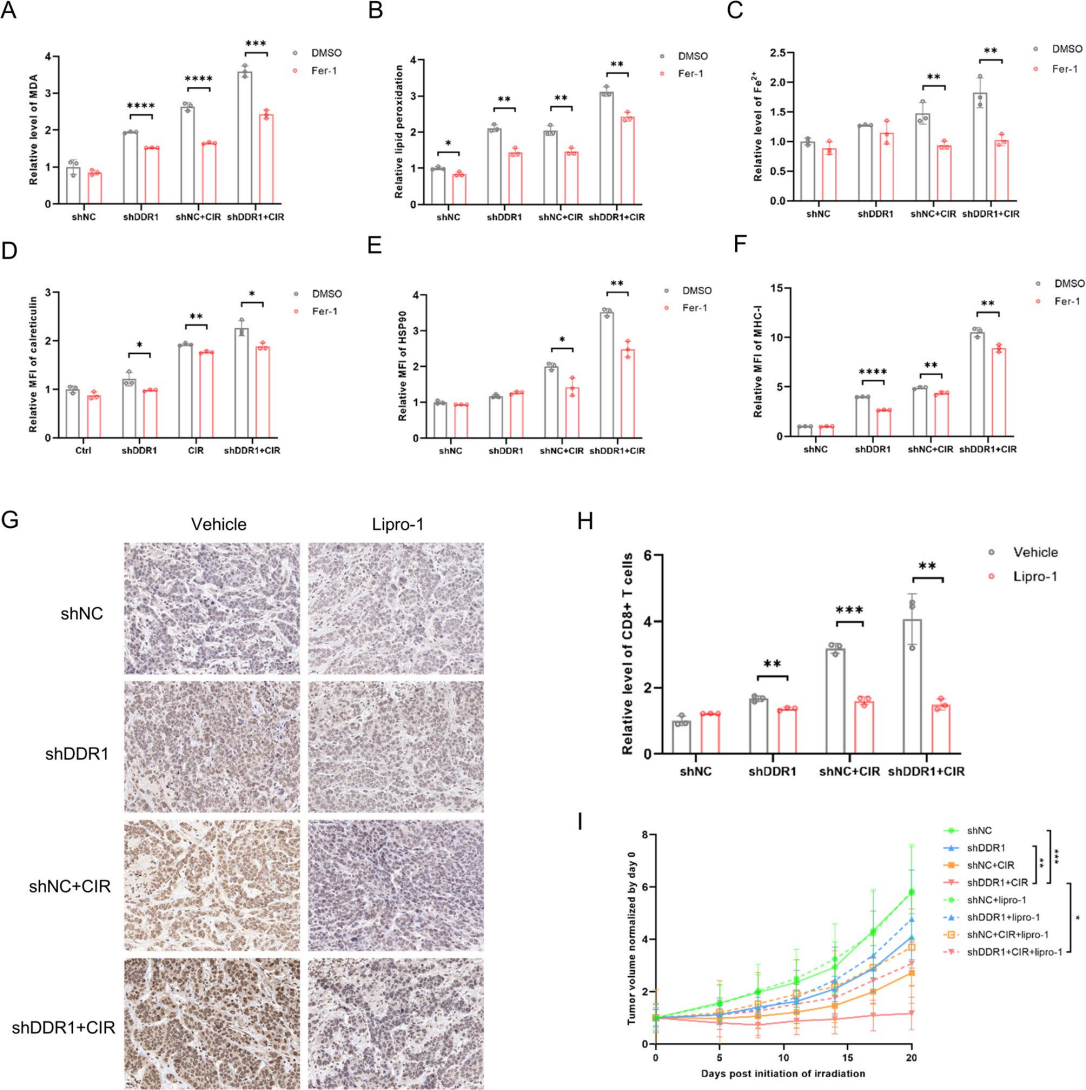
The ferroptosis pathway triggered by inhibiting DDR1 in conjunction with carbon ion radiotherapy bolsters antitumor immune responses. **A**-**C** Changes in MDA, lipid peroxidation, and Fe^2+^ levels in each group in MOC1 cells after Fer-1 treatment. **D**-**F** Changes in CRT, HSP90, and MHC-I expression levels in each group in MOC1 cells after Fer-1 treatment. **G** Immunohistochemistry staining of 4-HNE in mouse tumor tissues in each group with or without Lipro-1 treatment. Scale bar, 20 μm. **H** Changes in tumor-infiltrating CD8 + T cells in each group after Lipro-1 treatment. N = 3 mice/group. **I** Tumor growth in each group with or without Lipro-1 treatment. N = 5 mice/group. * *p* < 0.05, ** *p* < 0.01, *** *p* < 0.001, **** *p* < 0.0001

To further validate ferroptosis involvement in this therapeutic paradigm, we employed Fer-1 to inhibit ferroptosis at the cellular level. The results indicated that the elevations in MDA levels, lipid peroxidation, and Fe^2+^ levels observed in the shDDR1 + CIR group were effectively abrogated upon administration of the Fer-1 (Fig. 4A-C). Additionally, Fer-1 partially alleviated the significant enhancements in CRT, HSP90 and MHC-I levels caused by DDR1 inhibition and CIR (Fig. 4D-F). We then proceeded to extend our investigation to in vivo models, selecting Lipro-1 as a ferroptosis antagonist. Immunohistochemical analysis of 4-hydroxynonenal (4-HNE), a pivotal byproduct arising from lipid peroxidation, revealed marked elevation in the shDDR1 + CIR group, which was significantly mitigated by Lipro-1 co-treatment (Fig. 4G). Notably, Lipro-1 administration substantially diminished tumor-infiltrating CD8⁺ T lymphocyte populations across all treatment arms, with most pronounced effects observed in the shDDR1 + CIR cohort (Fig. 4H). Correspondingly, the antitumor efficacy of DDR1 inhibition combined with CIR was significantly compromised upon ferroptosis blockade (Fig. 4I). Collectively, these findings substantiated that ferroptosis activation served as a critical mechanism through which combined DDR1 suppression and CIR elicited immunogenic cell death in HNSCC.

### DDR1 modulates Akt/mTOR signaling via 14–3-3 interaction to affect carbon ion radiosensitivity

**Fig. 5:**
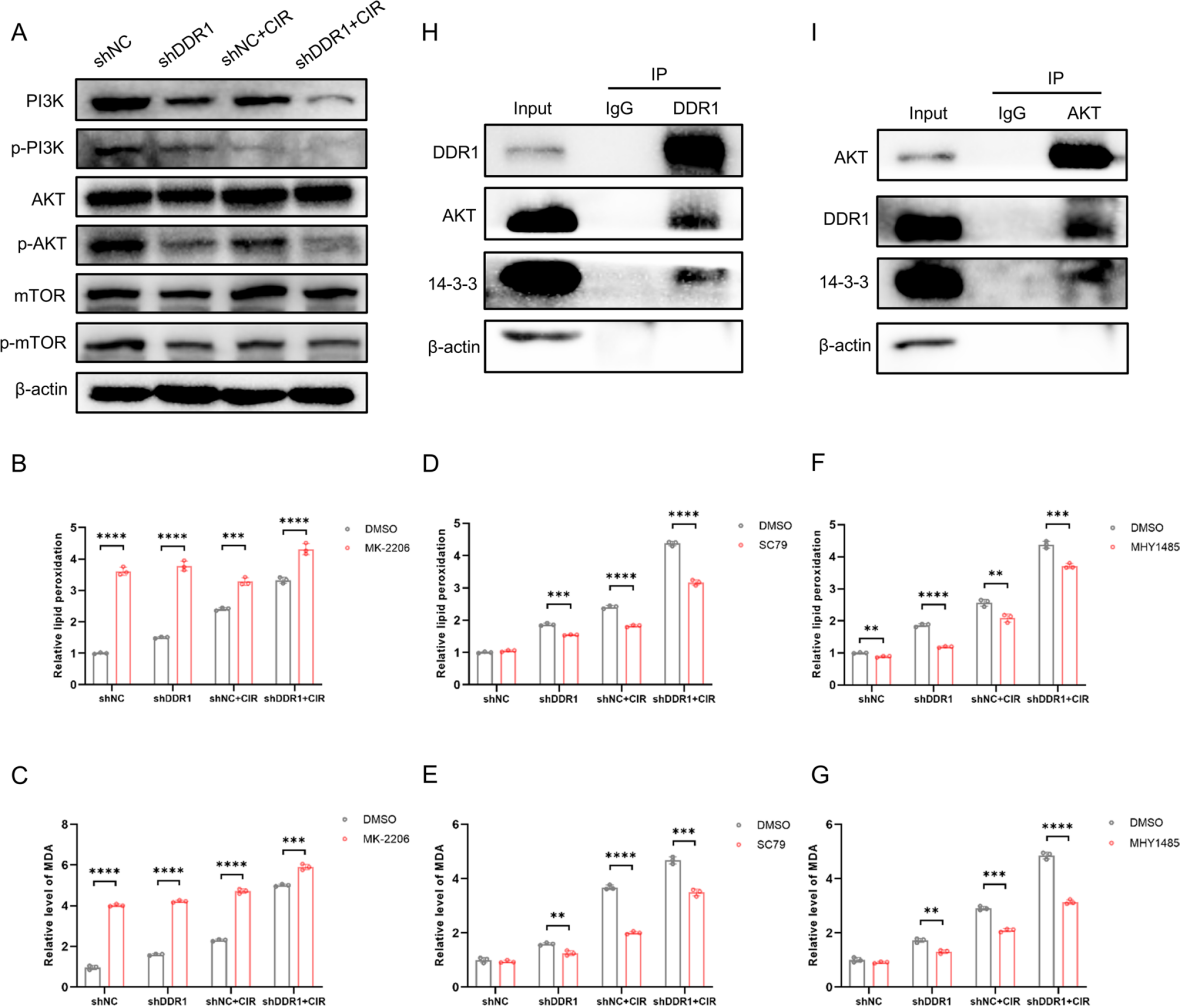
DDR1 regulates carbon ion radiosensitivity by affecting the Akt/mTOR pathway through 14–3-3. **A** Western blot analysis of protein expression levels involving PI3K/Akt/mTOR pathway in MOC1 cells after DDR1 knockdown and carbon ion radiotherapy. **B**–**G** Changes in lipid peroxidation and MDA levels in each group in MOC1 cells after treatment with (**B**, **C**) Akt inhibitor MK-2206, (**D**, **E**) Akt activator SC79 or (**F**, **G**) mTOR activator MHY1485. **H**, **I** Co-immunoprecipitation of endogenous DDR1, Akt and 14–3-3 from whole-cell lysates of MOC1 cells. ** *p* < 0.01, *** *p* < 0.001, **** *p* < 0.0001

We further explored the specific mechanism by which DDR1 inhibition combined with CIR induced ferroptosis. Referring back to our KEGG pathway enrichment analysis results, we identified a significant enrichment of the PI3K-Akt signaling pathway (Fig. 3A). Since mTOR is a key downstream effector molecule of the PI3K-Akt signaling pathway, we first verified through western blot experiments whether the PI3K/Akt/mTOR signaling pathway undergoes changes after DDR1 knockdown and CIR treatment. The results revealed substantial attenuation of PI3K (Tyr458), Akt (Ser473), and mTOR (Ser2448) phosphorylation following DDR1 knockdown and CIR exposure (Fig. 5A; Supplementary Fig. 7), suggesting inactivation of this pathway. To establish functional linkage between PI3K/Akt suppression and ferroptosis potentiation, we initially employed the Akt inhibitor MK-2206, and observed that MK-2206 exacerbated lipid peroxidation and MDA accumulation, especially in the control group (Fig. 5B, C). Conversely, the use of the Akt agonist SC79 effectively attenuated the elevation of lipid peroxidation and MDA levels caused by DDR1 inhibition and CIR (Fig. 5D, E). Also, complementary experiments with the mTORC1 activator MHY1485 demonstrated similar rescue effects, effectively reversing the high lipid peroxidation and MDA increases mediated by DDR1 inhibition and CIR (Fig. 5F, G). These findings demonstrated that PI3K/Akt/mTOR pathway inhibition serves as the principal mechanistic conduit through which DDR1 suppression and CIR drove ferroptosis.

To elucidate the DDR1-mediated regulation of carbon ion radiosensitivity via the Akt/mTOR axis, we hypothesized a potential protein-level interaction between DDR1 and Akt. Endogenous co-immunoprecipitation (Co-IP) assays in MOC1 cells confirmed DDR1-Akt binding (Fig. 5H). Given DDR1’s lack of canonical Akt-binding motifs, we investigated intermediary proteins, guided by prior evidence implicating 14–3-3 as a linker between DDR1 and Akt signaling [26]. Subsequent Co-IP validation demonstrated tripartite complex formation involving DDR1, 14–3-3, and Akt (Fig. 5H). Interestingly, Western blot analysis revealed a significant reduction in 14–3-3 protein levels following DDR1 knockdown and CIR treatment (Supplementary Fig. 8A). These findings establish a mechanistic axis wherein DDR1 engages Akt/mTOR signaling through 14–3-3, thereby modulating cellular response to carbon ion irradiation.

### Combined DDR1 inhibition and CIR induce ferroptosis by suppressing mTOR/SREBP1/SCD1-mediated lipogenesis

**Fig. 6:**
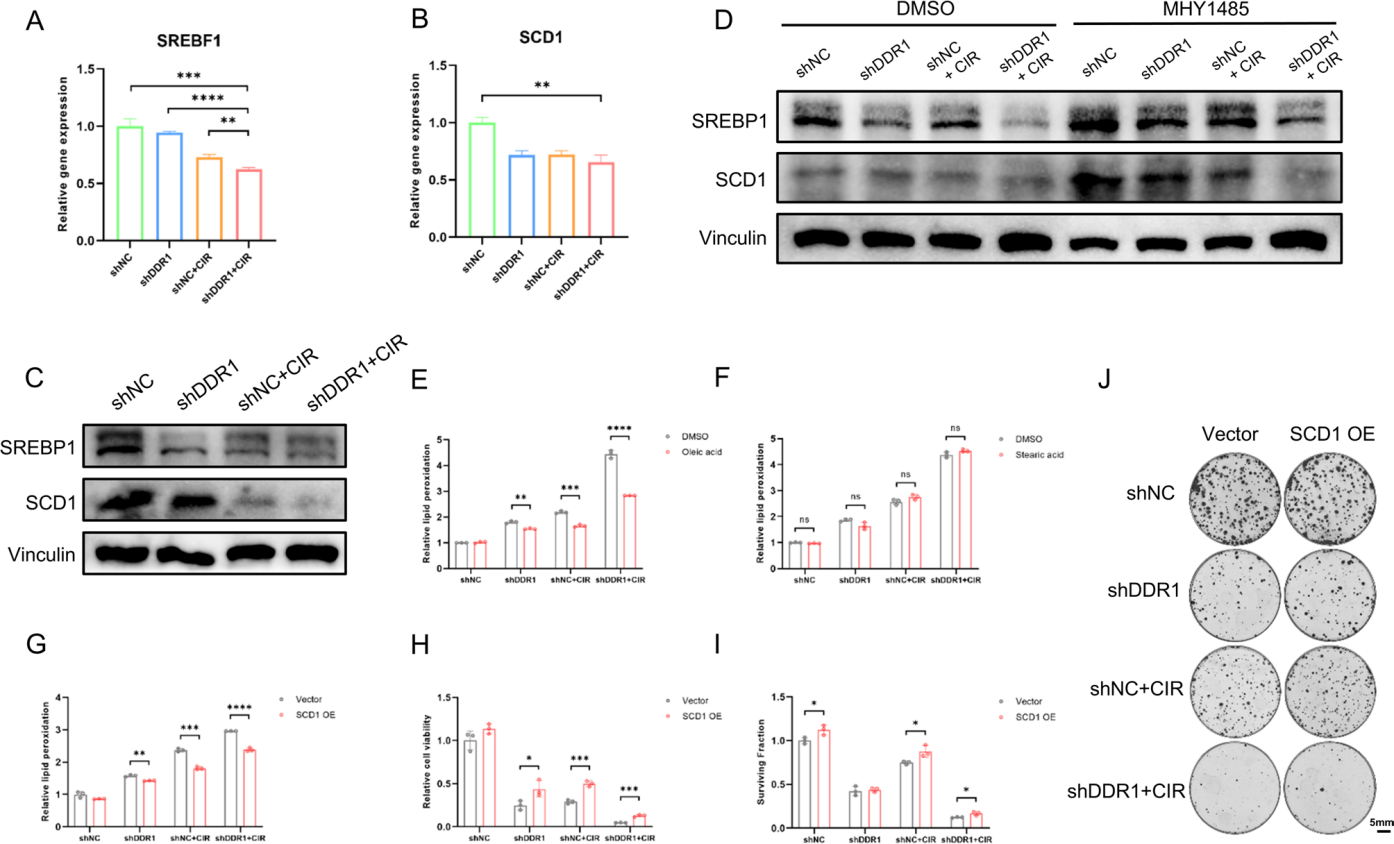
Combined DDR1 inhibition and CIR trigger ferroptosis by suppressing mTOR/SREBP1/SCD1-mediated lipogenesis. **A**, **B** qPCR analysis of SREBF1 and SCD1 mRNA levels in MOC1 cells after DDR1 knockdown and carbon ion radiotherapy. **C** Western blot analysis of SREBP1 and SCD1 protein levels in MOC1 cells after DDR1 knockdown and carbon ion radiotherapy. **D** Changes in SREBP1 and SCD1 protein expression levels in each group in MOC1 cells after MHY1485 treatment. **E**, **F** Changes in lipid peroxidation levels in each group in MOC1 cells after treatment with oleic acid and stearic acid. **G** Changes in lipid peroxidation levels in each group in MOC1 cells after overexpression of SCD1. **H** CCK-8 cell proliferation assay to detect cell viability after overexpression of SCD1 in MOC1 cells. **I**, **J** Colony formation assay to detect cell proliferation ability after overexpression of SCD1 in MOC1 cells, with representative images **J** and quantitative results **I**. ns non-significant, * *p* < 0.05, ** *p* < 0.01, *** *p* < 0.001, **** *p* < 0.0001

Building upon established evidence that PI3K/Akt/mTOR pathway inhibition potentiated ferroptosis via SREBP1/SCD1-mediated lipogenic regulation [27], we investigated whether DDR1 suppression combined with CIR modulated this metabolic axis to drive ferroptosis. We performed qPCR and western blot experiments, and the results revealed marked downregulation of SREBP1 and its downstream effector SCD1 following combination treatment (Fig. 6A-C). Furthermore, the mTOR activator MHY1485 rescued the decreased expression of SREBP1 and SCD1 induced by DDR1 knockdown and CIR (Fig. 6D). SCD1 is an essential lipid desaturase capable of converting saturated fatty acids (SFAs) into monounsaturated fatty acids (MUFAs) [28]. Therefore, we supplemented exogenous MUFA oleic acid (18:1, OA) and SFA stearic acid (18:0, SA), and discovered that unlike SA, the addition of OA could reduce the elevation of lipid peroxidation levels caused by DDR1 knockdown and CIR (Fig. 6E, F). To further confirm the role of SCD1 in ferroptosis, we overexpressed SCD1 in MOC1 cells and observed a significant decrease in cellular lipid peroxidation levels (Fig. 6G). Additionally, CCK-8 proliferation and colony formation assays indicated that SCD1 overexpression could enhanced cellular viability (Fig. 6H-J). In summary, these results indicated that inhibiting DDR1 combined with CIR promoted ferroptosis in HNSCC cells by suppressing mTOR/SREBP1/SCD1-mediated lipogenesis.

### DDR1 inhibitor 7rh enhances CIR sensitivity to suppress HNSCC tumor growth

**Fig. 7:**
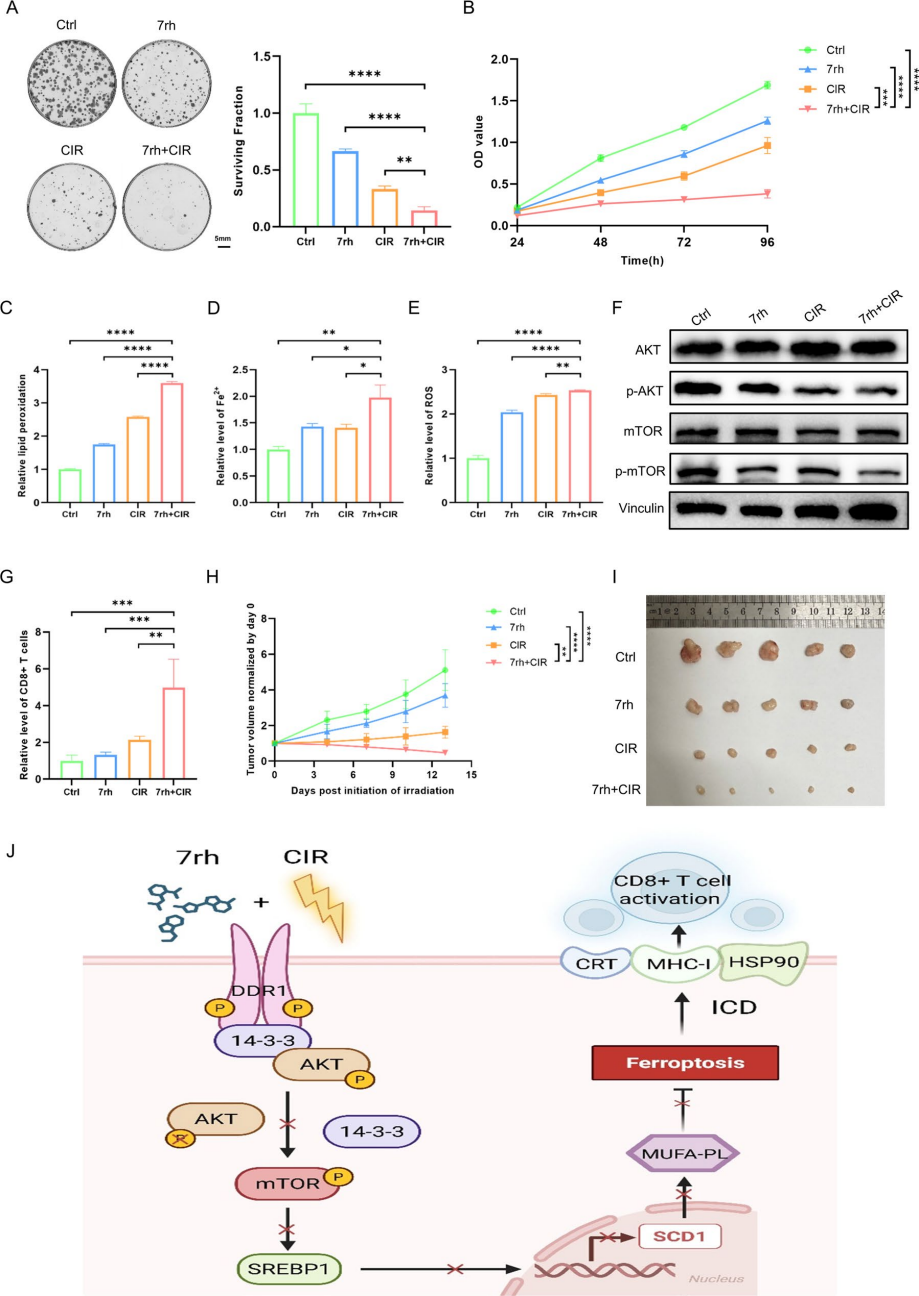
DDR1 inhibitor 7rh increases CIR sensitivity to suppress tumor growth of head and neck squamous cell carcinoma. **A** Representative images and quantitative results of colony formation assay in MOC1 cells after 7rh treatment and carbon ion radiotherapy. **B** CCK-8 cell proliferation assay in MOC1 cells after 7rh treatment and carbon ion radiotherapy. **C** BODIPY™ 581/591 C11 fluorescent probe to detect lipid peroxidation levels in MOC1 cells after 7rh treatment and carbon ion radiotherapy. **D** FerroOrange fluorescent probe to detect Fe^2+^ levels in MOC1 cells after 7rh treatment and carbon ion radiotherapy. **E** DCFH-DA fluorescent probe to detect ROS levels in MOC1 cells after 7rh treatment and carbon ion radiotherapy. **F** Western blot analysis of protein expression levels involving Akt/mTOR pathway in MOC1 cells after 7rh treatment and carbon ion radiotherapy. **G** Flow cytometry analysis of tumor-infiltrating CD8 + T cells in mice after treatment with 7rh and carbon ion radiotherapy. N = 5 mice/group. **H**, **I** Tumor growth and images of syngeneic MOC1 subcutaneous tumor models after treatment with 7rh and carbon ion radiotherapy. N = 5 mice/group. **J** Schematic diagram illustrating that the inhibition of DDR1 increases carbon ion radiosensitivity by inhibiting Akt/mTOR/SREBP1/SCD1-mediated lipogenesis of MUFAs, promoting ferroptosis and immunogenic cell death in head and neck squamous cell carcinoma. * *p* < 0.05, ** *p* < 0.01, *** *p* < 0.001, **** *p* < 0.0001

To enhance the clinical translational value of DDR1 as a therapeutic target for HNSCC, we employed a highly selective DDR1 inhibitor, 7rh, in combination with carbon ion therapy, aiming to observe its radiosensitization effect on HNSCC. Our results demonstrated that 7rh significantly suppressed HNSCC cell proliferation and enhanced radiosensitivity to carbon ion irradiation (Fig. 7A, B; Supplementary Fig. 9A, B). Mechanistically, 7rh treatment induced elevation of lipid peroxidation, Fe^2^⁺ accumulation, and ROS levels (Fig. 7C-E; Supplementary Fig. 9C-E), as well as reduction of PI3K (Tyr458), Akt (Ser473) and mTOR (Ser2448) phosphorylation and 14–3-3 levels (Fig. 7F; Supplementary Fig. 8B, 9F), pharmacologically confirming that DDR1 inhibition potentiated CIR by suppressing the Akt/mTOR pathway to promote ferroptosis, thereby sensitizing cells to radiotherapy. Furthermore, in vivo experiments revealed that the combination of 7rh and CIR exhibited remarkable increases in tumor-infiltrating CD8⁺ T lymphocytes (Fig. 7G) along with significant tumor growth suppression and regression trends (Fig. 7H, I). Collectively, these results demonstrated that 7rh could effectively promote ferroptosis in tumor cells and enhance the antitumor immune response induced by CIR in HNSCC.

## Discussion

HNSCC is a complex, highly heterogeneous malignancy. Despite radiotherapy being a cornerstone treatment, clinical outcomes remain suboptimal. CIR offers promise due to its unique physical and radiobiological benefits, including the induction of complex DNA damage and enhanced antitumor immunity [29, 30]. Although clinical studies [8–10] showed superior efficacy of CIR, limitations still persist, suggesting that CIR-induced immune activation may be offset by immunosuppressive mechanisms in HNSCC. Our study established DDR1 as a novel therapeutic target to amplify CIR efficacy by triggering ferroptosis-mediated immunogenic cell death. As illustrated in Fig. 7J, we identified a critical molecular mechanism wherein DDR1 facilitated Akt/mTOR signaling pathway activation in HNSCC cells through 14–3-3-mediated complex formation. Both genetic silencing and pharmacological inhibition of DDR1 disrupted the DDR1/14–3-3/Akt ternary complex, effectively suppressing mTORC1-dependent regulation of SREBP1/SCD1. This metabolic reprogramming attenuated MUFA biosynthesis, thereby alleviating MUFA-mediated suppression of ferroptosis. The resultant enhancement of ferroptosis augmented tumor immunogenicity, promoting CD8 + T cell infiltration and potentiating CIR-mediated antitumor effects.

Bioinformatics analysis of TCGA-HNSC datasets revealed significant DDR1 overexpression in HNSCC patients, correlating with adverse prognosis and inverse associations with cytotoxic immune markers (CD8B, GZMB, IFNG and IL12B) and lymphocyte infiltration scores, suggesting that DDR1 played a key role in inducing progression and immune escape in HNSCC. These findings aligned with prior studies [23, 31–34] documenting the overexpression of DDR1 and its role in therapy resistance across multiple malignancies like colorectal, breast and lung cancer, indicating that there might be a conserved mechanism across malignancies and DDR1 had the potential to be a cancer target. Our data also echoed the study [23] linking DDR1 to T cell exclusion in triple-negative breast cancer, although their proposed mechanism suggested that the extracellular domain of DDR1, rather than its intracellular kinase domain, aligned collagen fibers and then impeded immune infiltration. However, Su et al. [22] demonstrated no DDR1-dependent alterations in collagen architecture or CD8 + T cell infiltration in pancreatic cancer. This means that DDR1 may have different regulatory mechanisms in various tumors. Based on the role of DDR1 in HNSCC in tumor progression and immune escape, we believed that DDR1 could be a promising target for the combined application of CIR, thereby improving the therapeutic effect of HNSCC. This hypothesis was confirmed by the data collected in this study, which showed that DDR1 inhibition increased the radiosensitivity of CIR, specifically manifesting in the suppression of HNSCC cell proliferation and the delay of tumor growth in syngeneic MOC1 subcutaneous tumor models.

Preclinical and clinical data [11–14] indicated that the combination of radiotherapy and immunotherapy had promise for improving treatment effectiveness and reducing recurrence by increasing the capacity of the immune system to identify and eradicate tumor cells, and avoid tumor immune tolerance. Given that CIR had an immunomodulatory effect even superior to that of photon radiotherapy [16–19], CIR and immunotherapy were believed to have a synergistic effect. In fact, the combined therapeutic advantage of CIR and ICIs has been confirmed in preclinical studies in several tumors, including melanoma and breast cancer [35, 36]. Meanwhile, relevant multicenter clinical study is also underway [37]. Based on the immune regulatory role of DDR1, this study explored the efficacy of combining DDR1-targeted therapy with CIR. Surprisingly, the knockdown of DDR1 further augmented the tumor cell immunogenicity induced by CIR, accompanied by increased intratumoral CD8 + T cell infiltration, Granzyme B and IFN-γ production, indicating an enhancement in the killing capacity of immune cells. In addition, the expression of PD1 on tumor-infiltrating CD8 + T cells increased after CIR and rose further upon combined DDR1 inhibition. This suggests that PD1 expression could serve as a potential marker for tumor response, and combining anti-PD1 therapy holds promise for improving the treatment efficacy of HNSCC. We intend to continue exploring this research direction in the future.

A recent study [38] highlighted the pathological relevance of diverse programmed cell death ways in HNSCC development, including entosis, apoptosis, pyroptosis, autophagy, and ferroptosis. However, the predominant cell death mechanism governing therapeutic responses remains undefined. Ferroptosis is a novel form of programmed cell death, a type of cell death caused by iron-dependent lipid peroxidation and excessive ROS production. Previous studies [39–43] have demonstrated that ferroptosis served as a crucial mechanism in the carcinogenesis of HNSCC, influencing the treatment efficacy, including radiotherapy. Song et al. [42] found that radiotherapy promoted ferroptosis to exert tumor control effects in HNSCC by disrupting iron homeostasis, while glutamine blockade augmented this efficacy. Feng et al. [44] demonstrated that SLC7A11-mediated ferroptosis inhibition induced NRF2-associated radioresistance in esophageal squamous cell carcinoma. However, given the research gap regarding how DDR1 regulates ferroptosis and thereby affects the radiosensitivity of carbon ions in the fight against HNSCC, this study regarded ferroptosis as a clue to explore the potential molecular mechanisms by which inhibiting DDR1 might increase carbon ion radiosensitivity and promote immunogenic cell death in HNSCC. As expected, we demonstrated that DDR1 inhibition amplified multiple hallmarks of CIR-induced ferroptosis: intracellular Fe^2^⁺ accumulation, ROS elevation, lipid peroxidation, and characteristic mitochondrial ultrastructural changes. This ferroptotic cascade promoted immunogenic cell death, correlating with enhanced antitumor immunity, a phenomenon consistent with a recent study [45] linking ferroptosis to immunogenic death, which was related to endoplasmic reticulum stress.

The PI3K/Akt/mTOR signaling axis serves as a critical regulator of cellular homeostasis, governing fundamental processes including growth, proliferation, and metabolic programming. Its dysregulation is frequently implicated in tumorigenesis across various malignancies [46]. In HNSCC, this pathway emerges as a central oncogenic driver, promoting tumor progression, metastatic dissemination, and therapeutic resistance. Consequently, targeted inhibition of PI3K/Akt/mTOR signaling has gained recognition as a promising radiosensitization strategy in HNSCC management [47]. Emerging evidence across multiple tumor models has established an inverse regulatory relationship between PI3K/Akt/mTOR activation and ferroptosis susceptibility [27, 48]. Our findings corroborated this paradigm, demonstrating that DDR1 inhibition potentiated ferroptosis through suppression of PI3K/Akt/mTOR signaling, thereby enhancing therapeutic efficacy of CIR. The ferroptosis-enhancing effects were markedly amplified by co-treatment with the Akt inhibitor MK-2206, while pharmacological activation of Akt (SC79) or mTOR (MHY1485) effectively rescued HNSCC cells from DDR1 knockdown/CIR-induced ferroptosis. These complementary pharmacological interventions confirmed the pathway’s pivotal role in modulating ferroptosis dynamics. Mechanistic interrogation revealed that DDR1 engaged with Akt through 14–3-3 scaffold proteins to facilitate mTOR activation, thereby orchestrating downstream regulation of both ferroptotic sensitivity and radiation response. This molecular architecture aligned with previous reports by Vehlow et al. [26, 49] describing DDR1’s participation in a 14–3-3-BECN1-Akt1 complex that potentiated pro-survival signaling through Akt-mTOR axis activation. Collectively, our experimental data established PI3K/Akt/mTOR pathway inhibition as the principal mechanism underlying ferroptosis induction of combined DDR1 blockade and CIR. The 14–3-3 protein emerged as a critical molecular interface mediating DDR1-Akt crosstalk, presenting novel opportunities for therapeutic targeting in radiation oncology.

Ferroptosis initiation and execution demonstrate profound metabolic coupling with lipid dynamics, particularly through the intricate balance of PUFAs and MUFAs [50, 51]. The peroxidation of PUFA-PLs by radical species induces catastrophic membrane destabilization, constituting a biochemical hallmark of ferroptotic death [51, 52]. Contrastingly, MUFAs exhibit ferroptosis-suppressive activity through dual mechanisms: ROS scavenging capacity and competitive displacement of PUFAs from membrane phospholipids, with exogenous MUFA supplementation establishing ferroptosis-resistant cellular states [53]. The mTORC1 downstream effector SREBP1, a master transcriptional regulator of lipid biosynthesis, has emerged as both oncogenic driver and prognostic biomarker across malignancies [54]. Mechanistically, SREBP1 orchestrated fatty acid metabolic reprogramming through transcriptional activation of SCD1 [55], a pivotal enzyme converting SFAs to cytoprotective MUFAs that confer ferroptosis resistance [28].

Multiple analyses showed that SCD1 overexpression was associated with unfavorable outcomes in various tumor types [56–58]. Our experimental data corroborated these findings, demonstrating that combined DDR1 inhibition and CIR effectively suppressed the SREBP1-SCD1 axis to potentiate ferroptosis. This pro-death phenotype was reversed through either SCD1 overexpression or exogenous OA supplementation, confirming the critical anti-ferroptotic role of SCD1 and MUFA biosynthesis in HNSCC survival. Importantly, we identified mTORC1 signaling as the regulatory nexus coordinating this metabolic process, as the mTOR agonist MHY1485 effectively rescued the suppression of SREBP1/SCD1 protein expression induced by DDR1 knockdown and CIR. These findings positioned the PI3K/Akt/mTOR pathway as an essential regulator of SREBP1-mediated MUFA lipogenesis, with combinatorial DDR1 inhibition and CIR disrupting this pro-survival metabolic circuit to drive ferroptotic elimination of tumor cells.

Our findings established DDR1 as a promising therapeutic target for HNSCC, particularly for enhancing CIR efficacy. Based on this, a highly selective DDR1 inhibitor 7rh was employed to investigate its ability to sensitize tumor cells to CIR. This inhibitor has demonstrated significant therapeutic potential in various human cancer models, including pancreatic cancer, non-small cell lung cancer, and gastric cancer [59–61]. Our results indicated that 7rh effectively inhibited tumor growth both in vitro and in vivo, and promoted ferroptosis. Notably, 7rh combined with CIR significantly augmented CD8 + T cell tumor infiltration, suggesting dual modulation of ferroptotic death and antitumor immunity. These findings provided a compelling rationale for clinical trials evaluating 7rh and CIR combination therapy in HNSCC.

While mechanistically insightful, this work has three key limitations. First, clinical validation of DDR1 inhibition combined with CIR remains pending, and such trials would critically bridge preclinical and translational research. Second, single-cell profiling could further elucidate microenvironmental remodeling by DDR1 blockade and CIR. Third, given that DDR1 knockdown attenuated tumor invasion and migration, our focus on survival/proliferation mechanisms warrants expanded investigations into DDR1’s role in metastatic regulation. Addressing these gaps will be prioritized in subsequent studies.

In summary, this work positioned DDR1 targeting as a novel therapeutic strategy to potentiate CIR in HNSCC through immunogenic ferroptosis induction. Our findings advocate for clinical exploration of DDR1 inhibitors as radiosensitizing adjuvants, particularly for refractory or recurrent HNSCC where conventional therapies fail.

## Supplementary Information

Below is the link to the electronic supplementary material.

Supplementary Material 1

Supplementary Material 2

## Data Availability

All data generated or analysed during this study are available from the corresponding author on reasonable request.

## Abbreviations

4-HNE: 4-Hydroxynonenal
AO/PI: Acridine orange/propidium iodide
AUC: Area under the curve
CIR: Carbon ion radiotherapy
CRT: Calreticulin
DAMP: Damage-associated molecular pattern
DDR1: Discoidin domain receptor 1
DSB: Double-strand break
EGF: Epidermal growth factor
EMT: Epithelial-mesenchymal transition
FBS: Fetal bovine serum
Fer-1: Ferrostatin-1
HNSCC: Head and neck squamous cell carcinoma
HSP90: Heat shock protein 90
ICD: Immunogenic cell death
ICI: Immune checkpoint inhibitor
IHC: Immunohistochemistry
LET: Linear energy transfer
Lipro-1: Liproxstatin-1
MDA: Malondialdehyde
MUFA: Monounsaturated fatty acid
OA: Oleic acid
PE: Phosphatidylethanolamines
PUFA-PL: Polyunsaturated fatty acid-containing phospholipid
ROS: Reactive oxygen species
RT: Radiotherapy
SA: Stearic acid
SFA: Saturated fatty acid
SPHIC: Shanghai Proton and Heavy Ion Center
TME: Tumor microenvironment
