# Supplementary material for: Inhibition of DDR1 potentiates carbon ion radiotherapy by promoting ferroptosis and immunogenic death in head and neck squamous cell carcinoma
Source: J Transl Med. 2025 Sep 24;23:1011. doi: 10.1186/s12967-025-07062-5 (PMC12461992; doi:10.1186/s12967-025-07062-5)
Supplement: Supplementary file 1 — Supplementary Material 1 [file 12967_2025_7062_MOESM1_ESM.docx]

**Supplementary materials and methods**

**Data acquisition**

RNA-seq profiles and corresponding clinical records were acquired from the TCGA database. Following data integration and deduplication procedures, the final cohort comprised 546 subjects (44 normal controls; 502 tumor cases) with transcriptomic data.

**Lentivirus infection and plasmid transfection**

The lentiviral particles for DDR1 knockdown were obtained from Ubigene Biosciences (Guangzhou, China), while The SCD1 overexpression plasmid was purchased from Shanghai GeneChem Co., Ltd (Shanghai, China). For lentivirus infection, cells were transduced with lentivirus in the presence of polybrene (8 μg/mL) and selected with puromycin (2 μg/mL) for 3 days to establish stable knockdown lines. Knockdown efficiency was validated by qRT-PCR and Western blotting. For plasmid transfection, cells were seeded to achieve 70-90% confluency at the time of transfection. Plasmid DNA was diluted in Opti-MEM medium (Thermo Fisher Scientific) and gently mixed with Lipofectamine 2000 (Thermo Fisher Scientific) pre-diluted in Opti-MEM medium. The DNA-lipid complexes were incubated for 15 minutes at room temperature before being added dropwise to the cell culture. Transfected cells were harvested for downstream analyses 24-48 hours post-transfection. Overexpression efficiency was also validated by qRT-PCR and Western blotting.

**Colony formation assay**

Tumor cells were seeded in 6-well plates in triplicate at appropriate cell densities and allowed to adhere overnight. Following treatment administration, cells were cultured for 7-9 days at 37 °C and 5% CO_2_. Colonies were fixed with 4% paraformaldehyde after washing, stained with crystal violet, and quantified using an automated colony counter (GelCount^TM^ system, Oxford Optronix, UK) with a size threshold of ≥50 cells per colony. Survival fractions were normalized to untreated controls based on plating efficiency. Three independent experiments were performed.

**Cell viability assay**

Cell viability was assessed using the Cell Counting Kit-8 (CCK-8, Dojindo, Japan). Cells (2×10^3^/well) were plated in 96-well plates (n=5 replicates/group). At designated post-irradiation timepoints, culture medium was replaced with fresh medium containing 10% CCK-8 reagent. Following 1.5-2 h incubation at 37°C, absorbance was measured at 450 nm using a Cytation 3 microplate reader (BioTek, USA).

**Wound healing**

Cells were cultured in 6-well plates until reaching 90-95% confluency. A uniform wound was created in the monolayer using a sterile 200 μL pipette tip, followed by three washes with PBS to remove detached cells. Immediately after wounding, cells were subjected to irradiation. Images of the wound region were captured at baseline (0 h) and 12 h post-irradiation. The migration rate was calculated as: [(gap width at 0 h) - (gap width at 12 h)] / (gap width at 0 h) × 100.

**Transwell migration and invasion assays**

Cell migratory and invasive capacities were evaluated using 8 μm pore-size Transwell chambers (Corning Inc., USA). For migration assays, 6×10^4^ cells in serum-free medium were seeded into the upper chamber, while the lower chamber contained medium supplemented with 10% FBS. For invasion assays, the upper chamber membranes were pre-coated with Matrigel (1:8 dilution in serum-free medium; BD Biosciences) and incubated at 37°C for 2 hours to form a matrix barrier. After 36 hours (migration) or 48 hours (invasion) of incubation, non-migrated/invaded cells on the upper membrane surface were removed with a cotton swab. Cells that traversed the membrane were fixed with 4% paraformaldehyde, stained with crystal violet, and imaged under an inverted microscope.

**RNA sequencing and bioinformatics analysis**

Total RNA was extracted from cellular samples, followed by cDNA library preparation and transcriptome sequencing through BGI's commercial service (ID# F24A080000413_MUSashrN, Wuhan, China). Sequencing was performed on the DNBSEQ platform, with raw reads mapped against the mouse reference genome (Mus_musculus_10090.NCBI.GCF_000001635.26_GRCm38.p6.v2201). Functional enrichment analyses including Kyoto Encyclopedia of Genes and Genomes (KEGG) pathway profiling were conducted using BGI's proprietary Dr. TOM bioinformatics pipeline.

**Acridine Orange (AO) / Propidium Iodide (PI) staining**

After digestion, cells were resuspended in culture medium and mixed with AO/PI dye (BodBoge Technology, Guangzhou, China) at a 1:1 ratio. Following 5-min incubation at room temperature, 15 μL of the mixture was loaded into a counting chamber and analyzed using the manufacturer-matched automated fluorescence cell counter (BodBoge Technology, Guangzhou, China). This experiment was independently repeated three times.

**Lipid peroxidation assay**

Following 48-hour irradiation (with or without drug pretreatment), cells were trypsinized, washed twice with ice-cold PBS, and incubated with 5 μM BODIPY 581/591 C11 probe (Thermo Fisher Scientific) in serum-free medium at 37°C for 30 min. Fluorescence intensity were quantified using a CytoFLEX S flow cytometer (Beckman Coulter, USA) with 10,000 events recorded per sample. Data were derived from three independent biological experiments.

**Malondialdehyde (MDA) assay**

Following 48-hour irradiation (with or without drug pretreatment), cells were washed with ice-cold PBS, lysed in MDA lysis buffer, and centrifuged at 13,000 ×g for 10 min at 4°C. Supernatants were reacted with thiobarbituric acid (TBA) using a Lipid Peroxidation Assay Kit (Abcam) at 95°C for 60 min. After cooling, MDA-TBA adducts were quantified by measuring absorbance at OD 532 nm on a Cytation 3 microplate reader (BioTek, USA). MDA concentrations were normalized to total protein content (BCA assay). Data were derived from three independent biological replicates.

**Intracellular iron assay**

The level of intracellular Ferrous ion was determined were assessed via FerroOrange fluorescent probe (Dojindo, Japan). Cells were seeded and exposed to the indicated treatments. After treatment, cells were incubated with 1 µmol/L FerroOrange solution and incubated at 37 °C for 30 min. Fluorescence intensity was quantified using a CytoFLEX S flow cytometer (Beckman Coulter, USA), with 10,000 cellular events analyzed per sample. Data were derived from three independent biological replicates.

**Evaluation of reactive oxygen species (ROS) level**

Following seeding, cells were subjected to the specified experimental treatments. Following 48-hour irradiation, cells were loaded with 10 μM DCFH-DA (Sigma-Aldrich) in serum-free medium at 37°C for 30 min. After PBS washing, fluorescence intensity was quantified using a flow cytometer (Beckman Coulter, USA). Data were expressed as fold change versus controls (n=3 biological replicates).

**Transmission electron microscopy (TEM)**

After initial fixation in 2.5% glutaraldehyde (1 h, room temperature), cells were scraped, pelleted, and refixed in fresh 2.5% glutaraldehyde (24 h, 4°C). Post-fixation was performed with 1% osmium tetroxide (2 h, room temperature), followed by ethanol dehydration, acetone-epoxy resin infiltration, and epoxy resin embedding. Ultrathin sections (80-100 nm) were stained and imaged using a Hitachi HT7800 TEM (80 kV).

**Detection of** **immunogenicity**

Immunogenicity was assessed by measuring these biomarkers: calreticulin (CRT), MHC-I, HLA-ABC and HSP90. For calreticulin, MHC-I and HLA-ABC detection, cells were directly stained with PE-conjugated anti-CRT antibody (Abcam), APC-conjugated anti-MHC-I antibody (Invitrogen) and FITC-conjugated anti-HLA-ABC antibody (Invitrogen) for 1 h at 4°C in the dark. For HSP90 staining, cells were incubated with anti-HSP90 primary antibody (Selleck) under identical conditions, followed by CoraLite® Plus 488- conjugated secondary antibody (Proteintech) for 1 h at 4°C in the dark. Cells were washed twice and stained with DAPI Solution (BD Biosciences) for 5 min at room temperature. Samples were analyzed on a CytoFLEX S flow cytometer (Beckman Coulter, USA), with fluorescence quantified using FlowJo v10.8.1 software.

**Western blot**

Following cell lysis with RIPA buffer containing protease/phosphatase inhibitor cocktails (Epizyme), protein concentrations were quantified using a BCA assay kit (Epizyme). Lysates were denatured with 5× loading buffer at 95°C for 8 min, resolved via SDS-PAGE, and blotted onto PVDF membranes (Millipore). After blocking with 5% BSA/TBST for 1 h at room temperature, membranes were incubated overnight at 4°C with primary antibodies targeting: DDR1, E-Cadherin, Vimentin, ZEB1, Slug, PI3K, Phospho-PI3K (Tyr458), AKT, Phospho-AKT (Ser473), mTOR, Phospho-mTOR (Ser2448), GAPDH, β-actin, and Vinculin (all from Cell Signaling Technology); 14-3-3 (pan) and SREBP1 (Selleck); SCD1 (Proteintech). Membranes were washed, incubated with HRP-conjugated secondary antibody (Cell Signaling Technology) for 1 h at room temperature, and visualized using chemiluminescence with Image Lab software.

**Quantitative real-time PCR (qRT-PCR)**

Total RNA was extracted from MOC1 and Cal27 cells using SteadyPure Quick RNA Extraction Kit (Accurate Biology) in accordance with the manufacturer's instructions and quantified using a NanoDrop Lite spectrophotometer (Thermo Fisher Scientific). cDNA was prepared from 1 µg RNA using PrimeScript™ RT reagent Kit (Perfect Real Time) (Takara, Japan) and used for quantitative real-time PCR using TB Green® Premix Ex Taq™ (Tli RNaseH Plus) (Takara, Japan) by QuantStudio™ 5 Real-Time PCR System (Applied Biosystems). Primer sequences of mouse genes were as follows: SREBF1 forward, 5′- GATGTGCGAACTGGACACAG -3′; reverse, 5′- CATAGGGGGCGTCAAACAG -3′. SCD1 forward, 5′- TTCTTGCGATACACTCTGGTGC -3′; reverse, 5′- CGGGATTGAATGTTCTTGTCGT -3′. ACTB forward, 5′- GGCTGTATTCCCCTCCATCG -3′; reverse, 5′- CCAGTTGGTAACAATGCCATGT -3′. Primer sequences of human genes were as follows: HLA-A forward, 5′- GACGCCCCCAAAACGCATA -3′; reverse, 5′- TGGGCAAACCCTCATGCTG -3′. HLA-B forward, 5′- GGGATGGCGAGGACCAAAC -3′; reverse, 5′- ACAGCTCCGATGACCACAAC -3′. HLA-C forward, 5′- GACACAGAAGTACAAGCGCC -3′; reverse, 5′- TCGTAGGCGGACTGGTCATA -3′. ACTB forward, 5′- GGTGGCTTTTAGGATGGCAAG -3′; reverse, 5′- ACTGGAACGGTGAAGGTGACAG -3′. The relative mRNA levels were normalized based on the expression of ACTB. The relative expression of each gene was calculated using the 2^-ΔΔCT^ method.

**Untargeted Metabonomics and Lipidomics**

Untargeted metabolomic and lipidomic analysis were performed in SPHIC. Pre-treatment of cell samples followed the standard operation procedure. Briefly, 2 × 10⁶ cells were lysed in 400 μL methanol/water (1:1, v/v) through three freeze-thaw cycles in liquid nitrogen, followed by ice-cold sonication. Sequential lipid extraction was performed by adding 100 μL methanol and 200 μL methyl tert-butyl ether (MTBE), vortexed for 10 s, supplemented with an additional 190 μL MTBE and 160 μL water, then ultrasonicated in an ice-water bath (2 min). Phase separation was achieved via centrifugation (12,000 rpm, 15 min, 4°C), with 290 μL of the upper MTBE phase (lipid fraction) and 490 μL of the lower aqueous phase (metabolite fraction) collected separately. Solvents were evaporated using nitrogen blowing (lipid fraction) and vacuum centrifugation (metabolite fraction). Prior to LC-MS analysis, dried extracts were reconstituted in 80 μL solvent (acetonitrile/water = 3:1 for metabolomics; methanol/MTBE = 3:1 for lipidomics), vortexed (30 s), and ultrasonicated (5 min, ice-water bath). Post-centrifugation (12,000 rpm, 15 min, 4°C), supernatants were transferred to glass vials. Quality control (QC) samples were obtained by pooling equal volumes from all experimental samples and blank sample was generated by treating a blank tube with the above-mentioned methods.

The metabolomics data were collected by a UHPLC system (ExionLC™ 2.0, AB SCIEX, USA) coupled to a quadruple time-of-flight mass spectrometer (X500B, AB SCIEX, USA). Metabolites were separated using an ACQUITY UPLC BEH Amide Column (130Å, 1.7 µm, 2.1 mm × 100 mm; Waters, USA) in negative mode, while lipids were separated by a Kinetex C18 LC Column (100 Å, 2.6 µm, 100 × 2.1 mm; Phenomenex, USA) in the positive mode. Raw data (.wiff2/.wiff.scan) were centroided and converted to mzXML and mgf formats via ProteoWizard. The peaks relative quantitative matrixes of all samples were achieved through our proprietary R-based processing platform (Copyright ID: 2023SR0256527). Annotation pipelines were compound-class specific: metabolites were characterized using MetDNA2 (http://metdna.zhulab.cn/), and lipids were identified via Lipid4DAnalyzer (http://lipid4danalyzer.zhulab.cn/).

**Flow cytometry analysis of tumor-infiltrating CD8+ T cells**

Tumors harvested from euthanized mice were mechanically dissected and enzymatically digested in HBSS containing 1 mg/mL Collagenase D and 0.2 mg/mL DNase I (Roche) using the gentleMACS Octo Dissociator system (Miltenyi Biotec) with C Tubes. Erythrocytes were lysed using red blood cell lysing buffer (BD Pharmingen). Single-cell suspensions were stained in the dark (30 min, 4°C) with fluorophore-conjugated antibodies for cell surface markers: CD45-AF700, CD3-PE, CD4-APC, CD8-FITC (BD Pharmingen); PD-1-BV421 (BioLegend). Dead cells were excluded by DAPI counterstaining (5 min, room temperature) prior to flow cytometric analysis. For intracellular cytokine detection, cells were then incubated with Fixable Viability Stain 780 to label non-viable cells prior to fixation and permeabilization. Following incubation with surface-specific antibodies, cells were permeabilized using the Cytofix/Cytoperm™ Fixation/Permeabilization Kit (BD Pharmingen). Subsequently, intracellular staining was performed with BV421-conjugated IFN-γ antibody (BD Pharmingen) for 30 minutes at 4°C, followed by two washes. Flow cytometry was performed using CytoFLEX S (Beckman Coulter) and analyzed with CytExpert (Beckman Coulter) or FlowJo v10.8.1 software.

**Immunofluorescence**

Fresh tumor specimens were fixed in 4% paraformaldehyde for 24 h, embedded in paraffin, and sectioned. After being deparaffinized, rehydrated, and exposed to antigen retrieval, slides were blocked with 3% BSA and incubated with anti-CD4 and anti-CD8 primary antibodies overnight at 4°C in a humidified chamber. This is followed by species-matched FITC-conjugated and CY3-conjugated secondary antibodies for 1h at room temperature in the dark. Nuclei were counterstained with DAPI. Imaging was performed on a fluorescence microscope (Nikon 80i).

**Histology and immunohistochemistry (IHC) analysis**

Paraffin-embedded tumor tissues from C57BL/6 mice were sectioned and processed for histological analysis. Hematoxylin/eosin (H&E) staining was performed using standard protocols. For immunohistochemistry, sections underwent sequential deparaffinization, rehydration, and heat-induced antigen retrieval in sodium citrate buffer (pH 6.0). Endogenous peroxidase activity was inhibited with 3% H₂O₂, followed by blocking with 3% BSA. Primary antibodies against Ki67 (Abcam), 4-HNE (Abcam), and GZMB (Invitrogen) were applied overnight at 4°C, with HRP-conjugated secondary antibodies incubated for 1h at room temperature. Chromogenic detection was achieved using a DAB substrate kit, followed by hematoxylin counterstaining. Slides were imaged under a microscopy.

**Co-immunoprecipitation (Co-IP)**

Cell lysates were prepared using lysis buffer (Beyotime) containing protease/phosphatase inhibitors (Epizyme), centrifuged at 12,000 ×g (15 min, 4°C), and pre-cleared with Protein A/G Plus-Agarose beads (Thermo Fisher Scientific) plus 2 μg/mL species-matched IgG (Proteintech) (1 h, 4°C). Pre-cleared lysates were then incubated with fresh Protein A/G Plus-Agarose beads and target-specific antibodies (1:50-1:100 dilution) overnight at 4°C under gentle rotation. The bead-antibody-protein complexes were pelleted by centrifugation (2,500 ×g, 5 min) and washed five times with cold PBS. Bound proteins were eluted by boiling the beads in 2×SDS buffer at 95°C for 5 min. Co-precipitated proteins were resolved via SDS-PAGE and transferred to PVDF membranes for immunoblot analysis using validated primary antibodies. Antibody-antigen interactions were visualized with HRP-conjugated secondary antibodies and chemiluminescent substrate.
