# Supplementary material for: Inhibition of DDR1 potentiates carbon ion radiotherapy by promoting ferroptosis and immunogenic death in head and neck squamous cell carcinoma
Source: J Transl Med. 2025 Sep 24;23:1011. doi: 10.1186/s12967-025-07062-5 (PMC12461992; doi:10.1186/s12967-025-07062-5)
Supplement: Supplementary file 2 — Supplementary Material 2 [file 12967_2025_7062_MOESM2_ESM.docx]

**
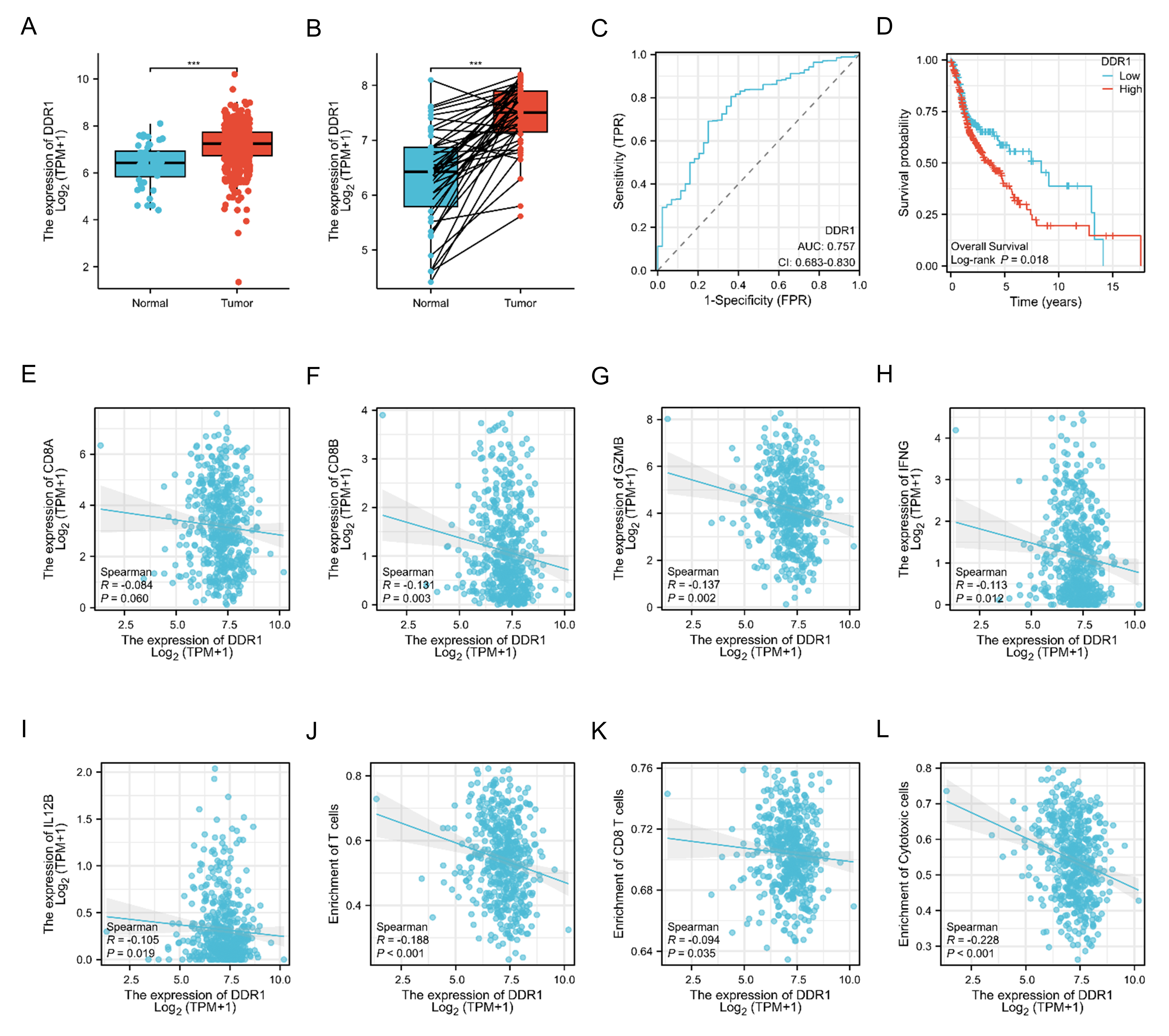
**

**Supplementary Fig. 1** Expression characteristic, prognosis value, and immune correlation of DDR1 in head and neck squamous cell carcinoma. (A) Comparative analysis of DDR1 levels in HNSCC tissues and normal tissues from the TCGA-HNSC dataset. (B) Comparative analysis of DDR1 levels in paired HNSCC tissues and normal tissues from the TCGA-HNSC dataset. (C) ROC curve indicating the predictive value of DDR1. (D) Comparison of the low and high expression of DDR1 via Kaplan–Meier OS curve from the TCGA-HNSC dataset. (E-I) Negative correlation between DDR1 expression and CD8A, CD8B, GZMB, IFNG and IL12B. (J-L) Negative correlation between DDR1 expression and scores for T cells, CD8 T cells, and cytotoxic cells. *** *p* < 0.001.


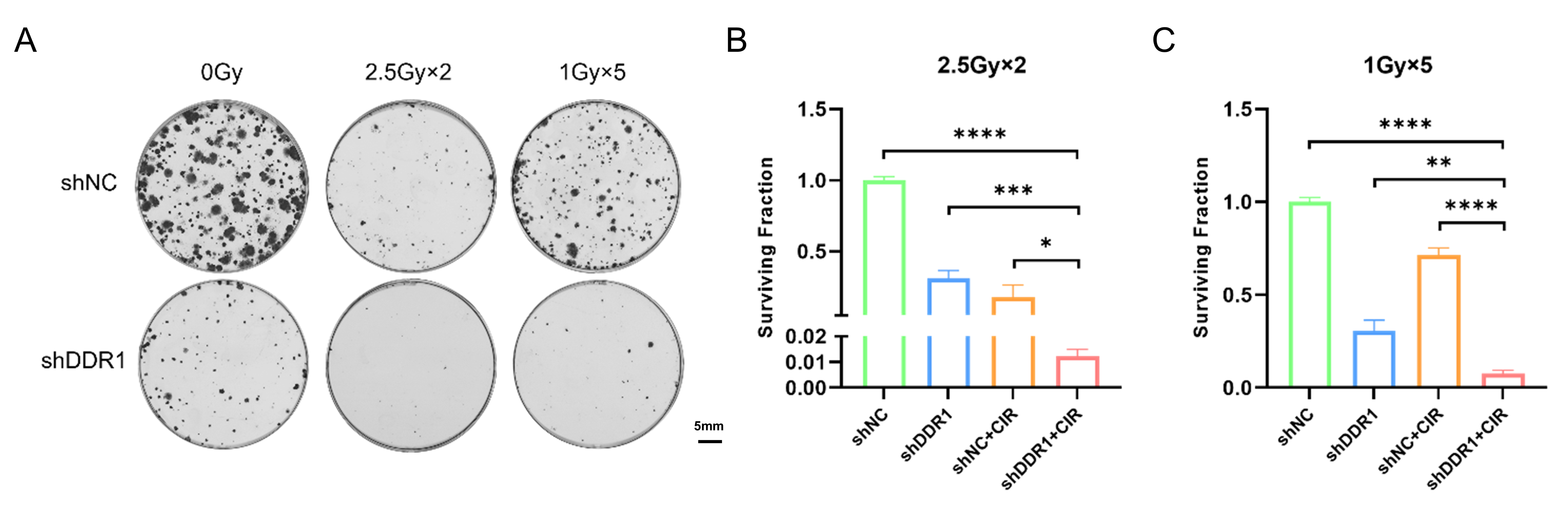


**Supplementary Fig. 2** Survival after treatment of DDR1 knockdown and fractionated carbon ion radiotherapy in MOC1 cells. (A-C) Representative images or quantitative results of colony formation assay in MOC1 cells after DDR1 knockdown and fractionated carbon ion radiotherapy. Scale bar, 5 mm. * p < 0.05, ** p < 0.01, *** p < 0.001, **** p < 0.0001.


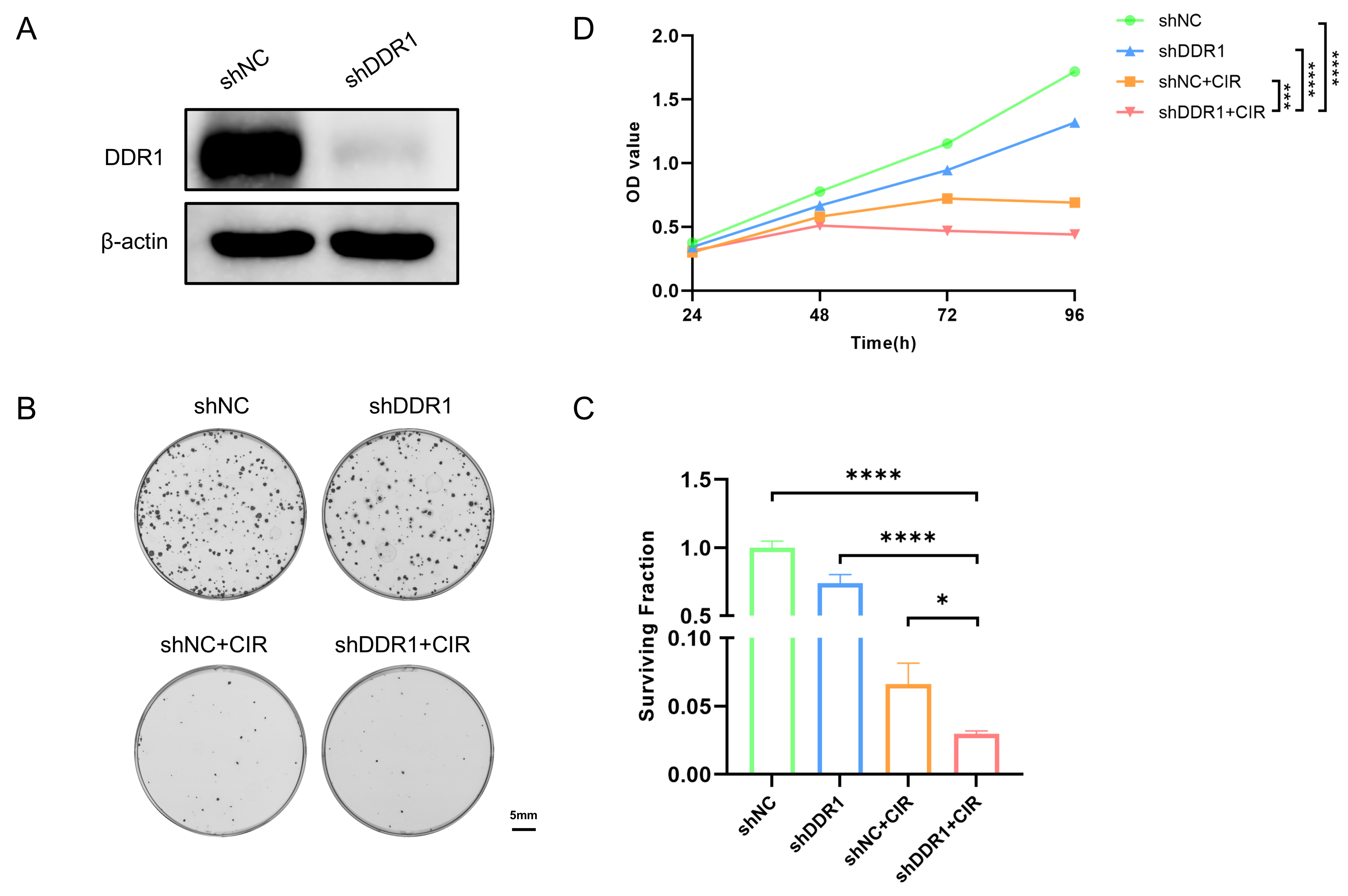


**Supplementary Fig. 3** Antitumor effect of DDR1 knockdown combined with carbon ion radiotherapy in Cal27 cells. (A) Cal27 cells were infected with lentiviral particles for DDR1 knockdown. Western blot analysis of DDR1 protein levels in DDR1-knockdown and control cells. (B, C) Representative images or quantitative results of colony formation assay in Cal27 cells after DDR1 knockdown and carbon ion radiotherapy. Scale bar, 5 mm. (D) CCK-8 cell proliferation assay in Cal27 cells after DDR1 knockdown and carbon ion radiotherapy. * p < 0.05, *** p < 0.001, **** p < 0.0001.


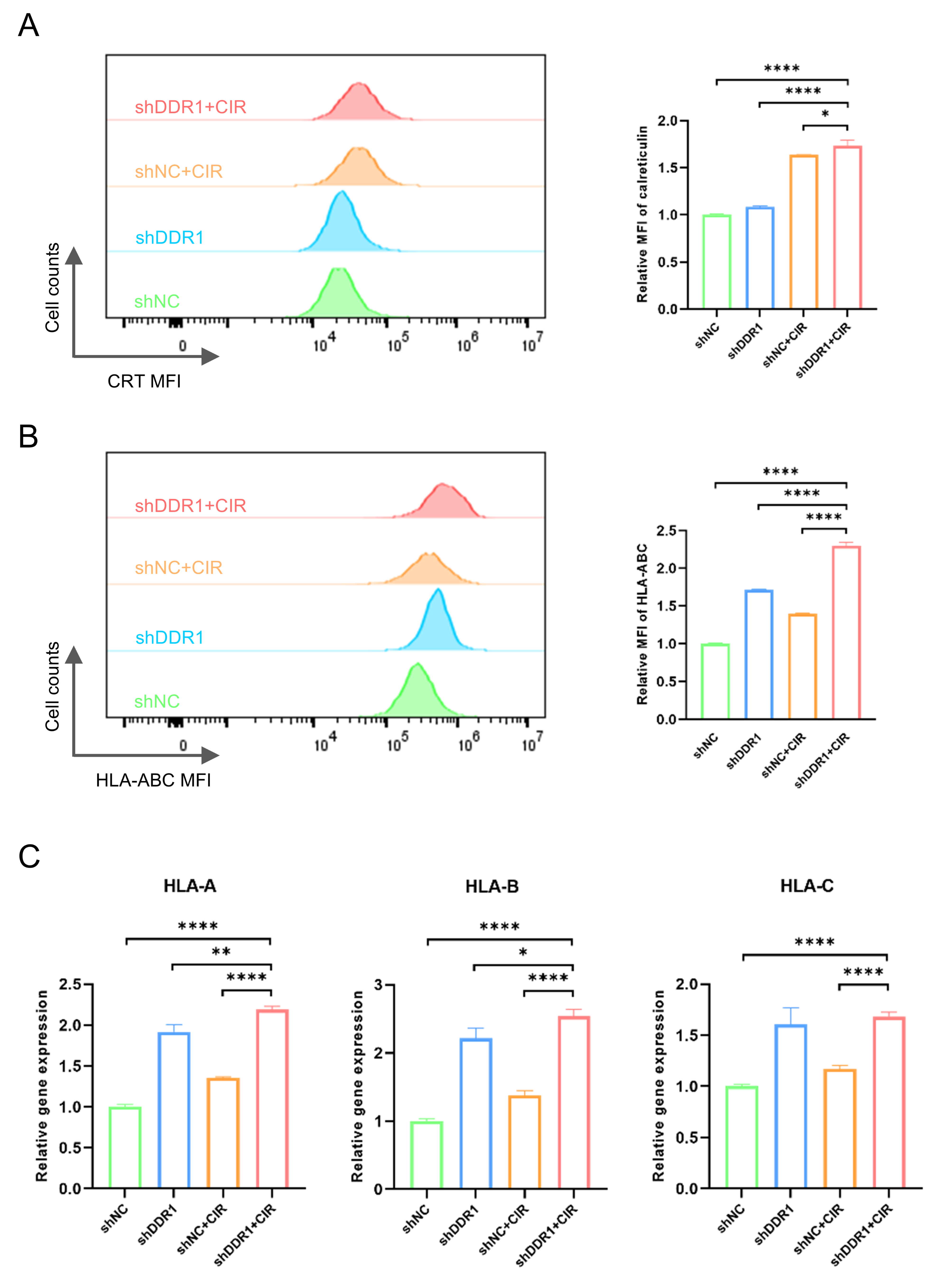


**Supplementary Fig. 4** Inhibition of DDR1 combined with carbon ion radiotherapy increases immunogenicity in Cal27 cells. (A, B) Flow cytometry analysis of CRT and HLA-ABC expression levels on Cal27 cells after DDR1 knockdown and carbon ion radiotherapy. (C) qPCR analysis of HLA-A, HLA-B and HLA-C mRNA levels in Cal27 cells after DDR1 knockdown and carbon ion radiotherapy. * p < 0.05, ** p < 0.01, **** p < 0.0001.


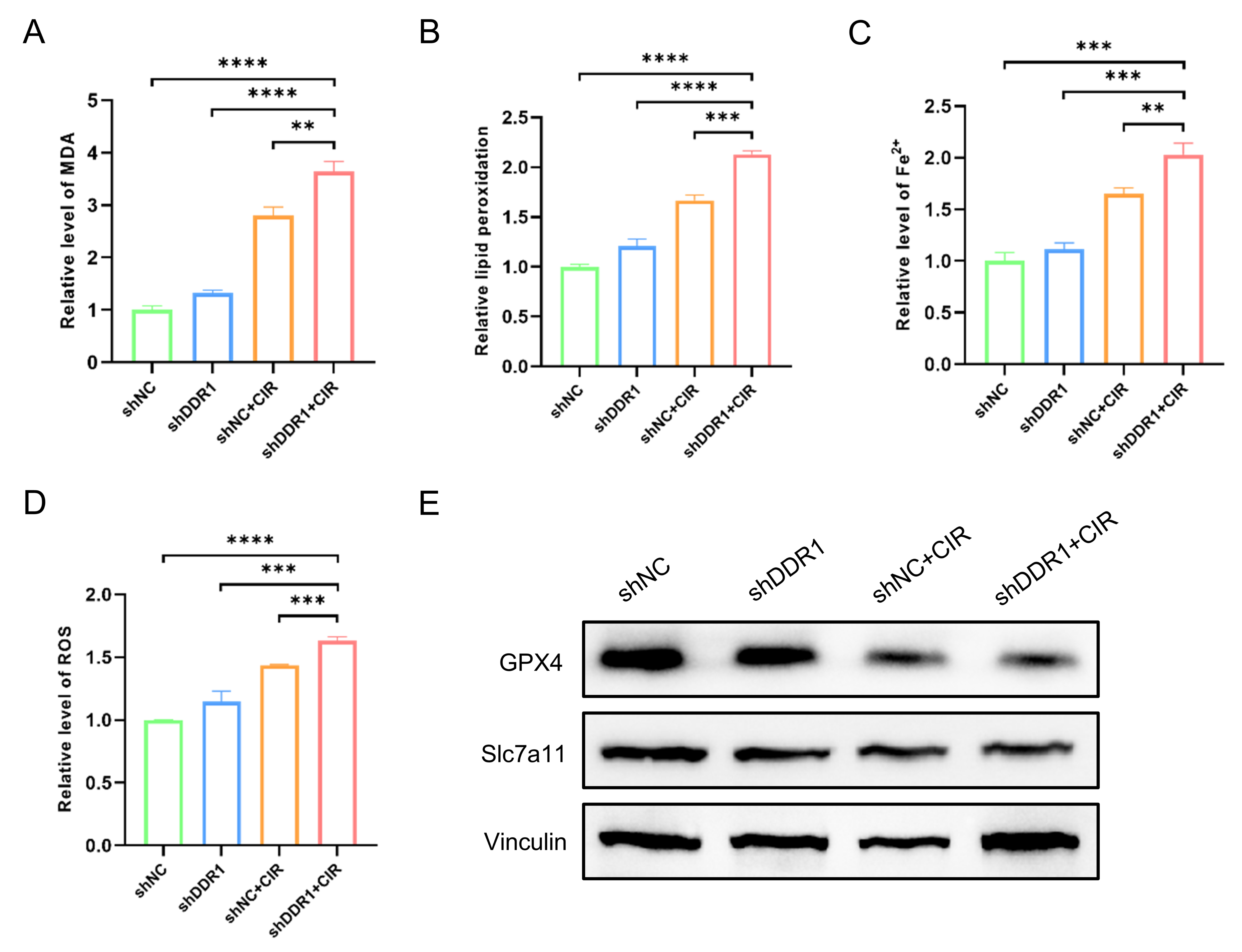


**Supplementary Fig. 5** Inhibition of DDR1 combined with carbon ion radiotherapy promotes ferroptosis in Cal27 cells. (A) MDA assay to measure lipid peroxidation levels in Cal27 cells after DDR1 knockdown and carbon ion radiotherapy. (B) BODIPY™ 581/591 C11 fluorescent probe to detect lipid peroxidation levels in Cal27 cells after DDR1 knockdown and carbon ion radiotherapy. (C) FerroOrange fluorescent probe to detect Fe²⁺ levels in Cal27 cells after DDR1 knockdown and carbon ion radiotherapy. (D) DCFH-DA fluorescent probe to detect ROS levels in Cal27 cells after DDR1 knockdown and carbon ion radiotherapy. (E) Western blot analysis of GPX4 and SLC7A11 expression in Cal27 cells after DDR1 knockdown and carbon ion radiotherapy. ** p < 0.01, *** p < 0.001, **** p < 0.0001.


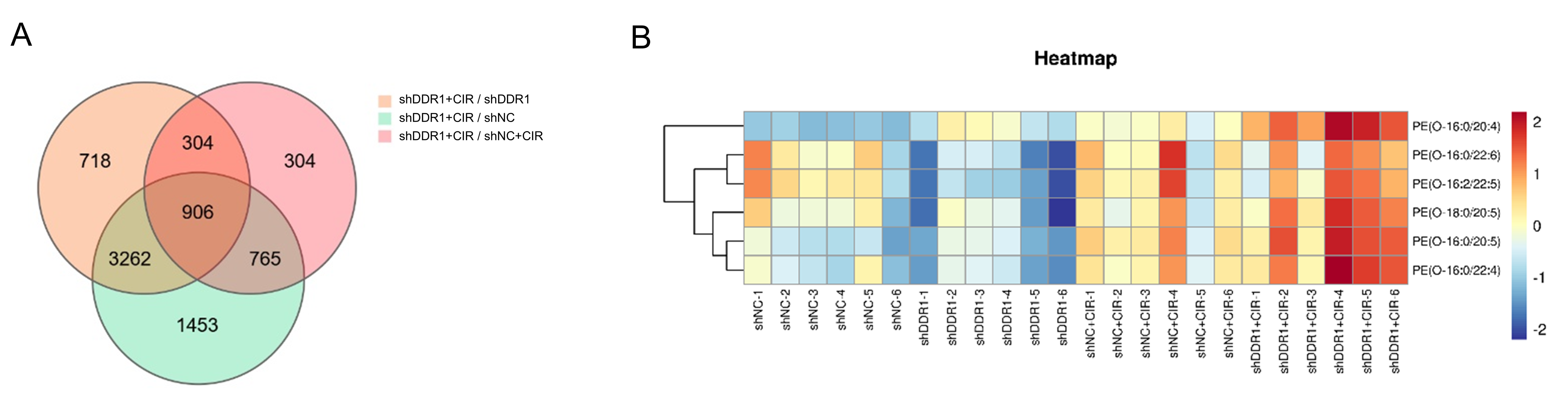


**Supplementary Fig. 6** Transcriptomics and untargeted metabolomics of MOC1 cells after DDR1 knockdown and carbon ion radiotherapy. (A) Venn diagram showing differentially expressed genes between the combined DDR1 knockdown and carbon ion radiotherapy group and other groups. (B) Heatmap of PUFA-PLs levels detected by untargeted lipidomics in MOC1 cells after DDR1 knockdown and carbon ion radiotherapy.


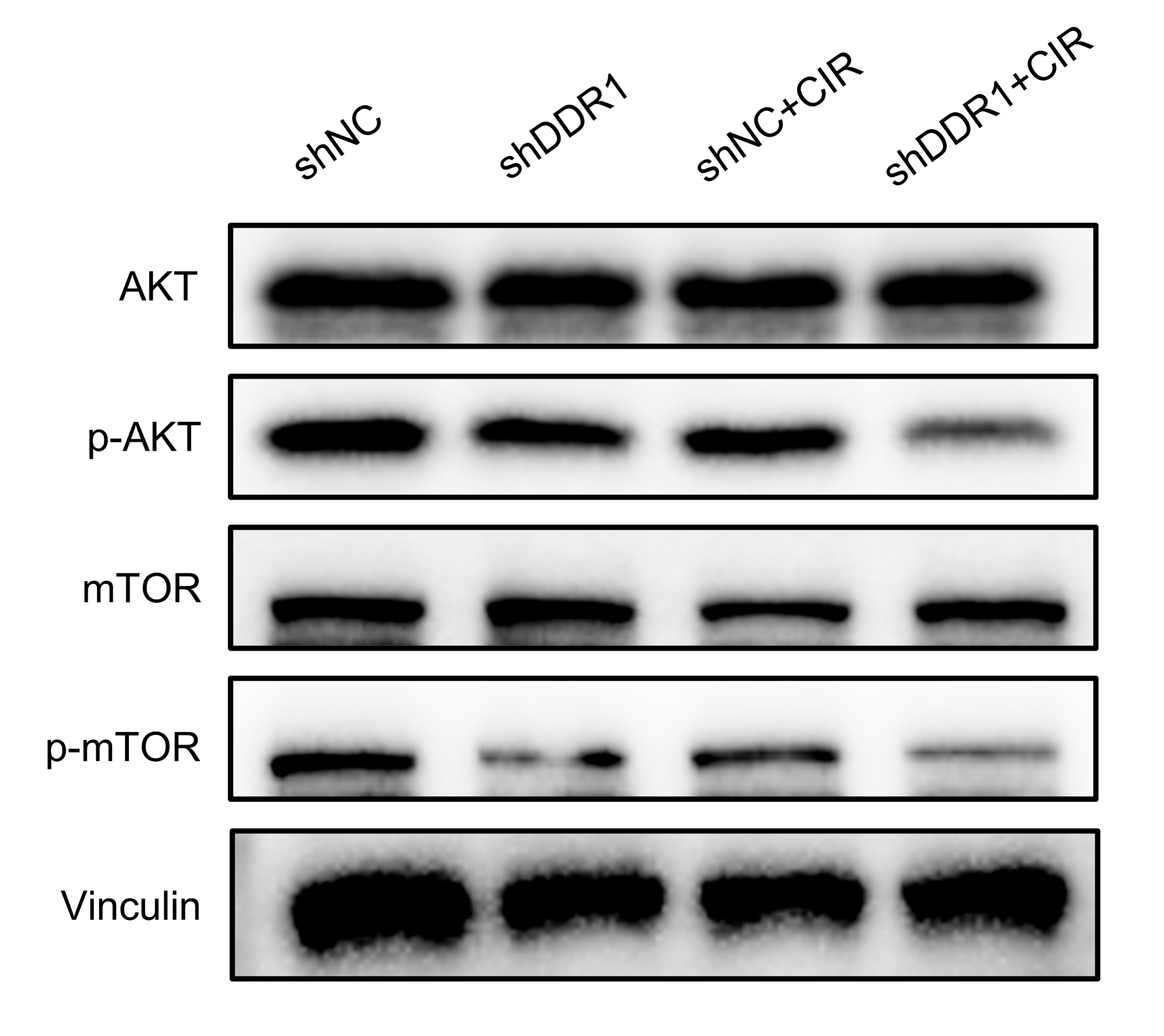


**Supplementary Fig. 7** Western blot analysis of protein expression levels involving Akt/mTOR pathway in Cal27 cells after DDR1 knockdown and carbon ion radiotherapy.


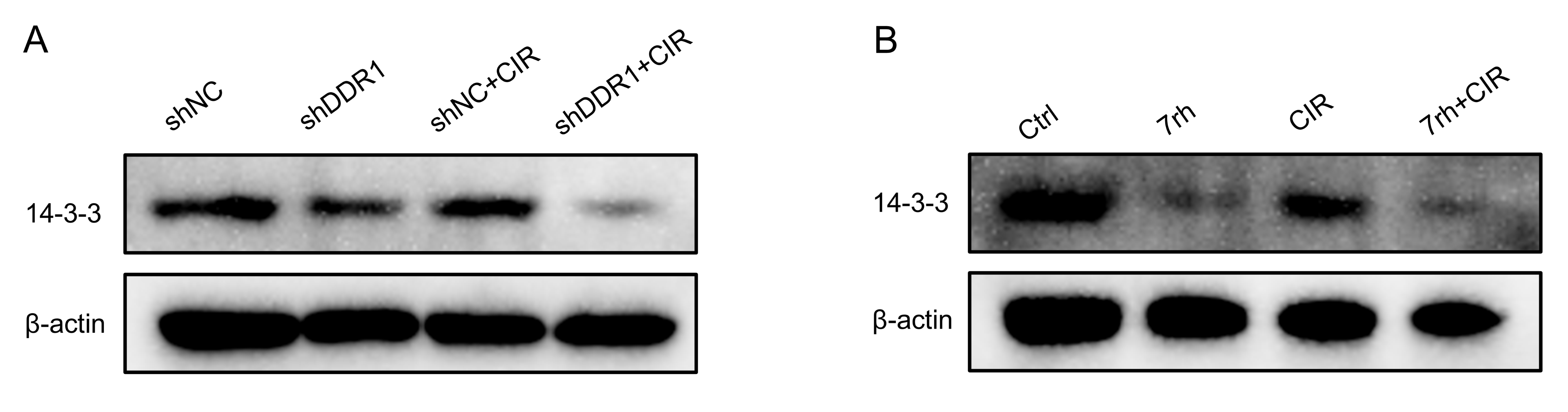


**Supplementary Fig. 8** 14-3-3 protein expression level in MOC1 cells after DDR1 inhibition and carbon ion radiotherapy. (A) Western blot analysis of 14-3-3 protein expression level in MOC1 cells after DDR1 knockdown and carbon ion radiotherapy. (B) Western blot analysis of 14-3-3 protein expression level in MOC1 cells after 7rh treatment and carbon ion radiotherapy.


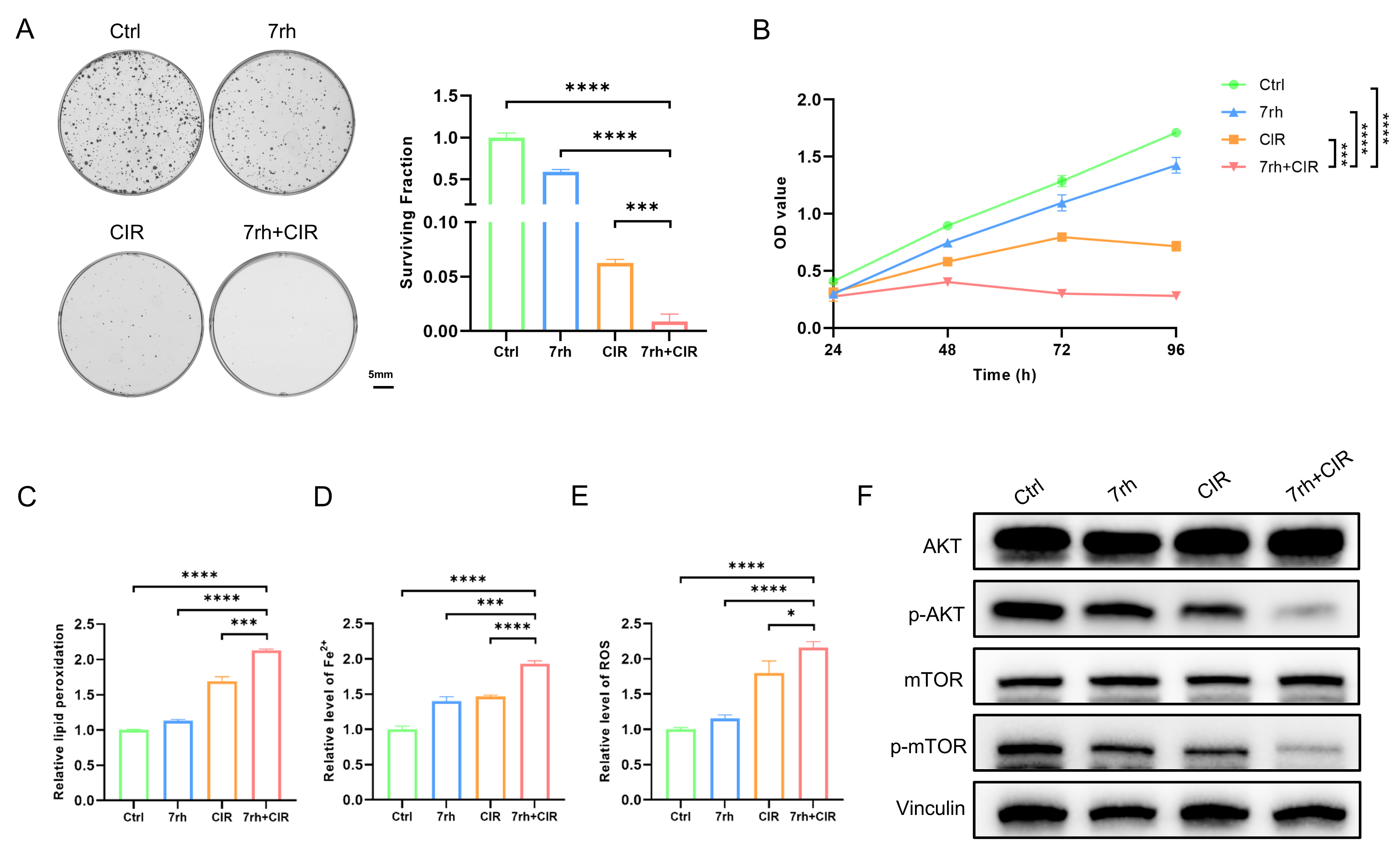


**Supplementary Fig. 9** DDR1 inhibitor 7rh increases ferroptosis and CIR sensitivity in Cal27 cells. (A) Representative images and quantitative results of colony formation assay in Cal27 cells after 7rh treatment and carbon ion radiotherapy. (B) CCK-8 cell proliferation assay in Cal27 cells after 7rh treatment and carbon ion radiotherapy. (C) BODIPY™ 581/591 C11 fluorescent probe to detect lipid peroxidation levels in Cal27 cells after 7rh treatment and carbon ion radiotherapy. (D) FerroOrange fluorescent probe to detect Fe²⁺ levels in Cal27 cells after 7rh treatment and carbon ion radiotherapy. (E) DCFH-DA fluorescent probe to detect ROS levels in Cal27 cells after 7rh treatment and carbon ion radiotherapy. (F) Western blot analysis of protein expression levels involving Akt/mTOR pathway in Cal27 cells after 7rh treatment and carbon ion radiotherapy. * p < 0.05, *** p < 0.001, **** p < 0.0001.
